# Boundary-based registration improves sensitivity for detecting hypoperfusion in sporadic frontotemporal lobar degeneration

**DOI:** 10.3389/fneur.2024.1452944

**Published:** 2024-08-21

**Authors:** Sylvia Mihailescu, Quinn Hlava, Philip A. Cook, Maria Luisa Mandelli, Suzee E. Lee, Bradley F. Boeve, Bradford C. Dickerson, Maria Luisa Gorno-Tempini, Emily Rogalski, Murray Grossman, James Gee, Corey T. McMillan, Christopher A. Olm

**Affiliations:** ^1^School of Engineering and Applied Sciences, University of Pennsylvania, Philadelphia, PA, United States; ^2^Department of Neurology, University of Pennsylvania, Philadelphia, PA, United States; ^3^Department of Radiology, University of Pennsylvania, Philadelphia, PA, United States; ^4^Memory and Aging Center, Department of Neurology, Weill Institute for Neurosciences, University of California San Francisco, San Francisco, CA, United States; ^5^Department of Neurology, Mayo Clinic, Rochester, MN, United States; ^6^Department of Neurology, Massachusetts General Hospital, Boston, MA, United States; ^7^Healthy Aging & Alzheimer’s Care Center, University of Chicago, Chicago, IL, United States; ^8^Department of Neurology, University of Chicago, Chicago, IL, United States

**Keywords:** arterial spin labeling, image registration, frontotemporal lobar degeneration, cerebral blood flow, boundary-based registration

## Abstract

**Introduction:**

Frontotemporal lobar degeneration (FTLD) is associated with FTLD due to tau (FTLD-tau) or TDP (FTLD-TDP) inclusions found at autopsy. Arterial Spin Labeling (ASL) MRI is often acquired in the same session as a structural T1-weighted image (T1w), enabling detection of regional changes in cerebral blood flow (CBF). We hypothesize that ASL-T1w registration with more degrees of freedom using boundary-based registration (BBR) will better align ASL and T1w images and show increased sensitivity to regional hypoperfusion differences compared to manual registration in patient participants. We hypothesize that hypoperfusion will be associated with a clinical measure of disease severity, the FTLD-modified clinical dementia rating scale sum-of-boxes (FTLD-CDR).

**Materials and methods:**

Patients with sporadic likely FTLD-tau (sFTLD-tau; *N* = 21), with sporadic likely FTLD-TDP (sFTLD-TDP; *N* = 14), and controls (*N* = 50) were recruited from the Connectomic Imaging in Familial and Sporadic Frontotemporal Degeneration project (FTDHCP). Pearson’s Correlation Coefficients (CC) were calculated on cortical vertex-wise CBF between each participant for each of 3 registration methods: (1) manual registration, (2) BBR initialized with manual registration (manual+BBR), (3) and BBR initialized using FLIRT (FLIRT+BBR). Mean CBF was calculated in the same regions of interest (ROIs) for each registration method after image alignment. Paired *t*-tests of CC values for each registration method were performed to compare alignment. Mean CBF in each ROI was compared between groups using *t*-tests. Differences were considered significant at *p* < 0.05 (Bonferroni-corrected). We performed linear regression to relate FTLD-CDR to mean CBF in patients with sFTLD-tau and sFTLD-TDP, separately (*p* < 0.05, uncorrected).

**Results:**

All registration methods demonstrated significant hypoperfusion in frontal and temporal regions in each patient group relative to controls. All registration methods detected hypoperfusion in the left insular cortex, middle temporal gyrus, and temporal pole in sFTLD-TDP relative to sFTLD-tau. FTLD-CDR had an inverse association with CBF in right temporal and orbitofrontal ROIs in sFTLD-TDP. Manual+BBR performed similarly to FLIRT+BBR.

**Discussion:**

ASL is sensitive to distinct regions of hypoperfusion in patient participants relative to controls, and in patients with sFTLD-TDP relative to sFTLD-tau, and decreasing perfusion is associated with increasing disease severity, at least in sFTLD-TDP. BBR can register ASL-T1w images adequately for controls and patients.

## 1 Introduction

Frontotemporal lobar degeneration (FTLD) is a progressive neurodegenerative disorder that causes degeneration predominantly in the frontal and temporal lobes of the brain. Sporadic FTLD (sFTLD) is frequently a monoproteinopathy, most often due to either tau (FTLD-tau) or tar-DNA binding protein (FTLD-TDP) (1, 2). sFTLD presents in a number of syndromes that may involve both cognitive and motor impairments, such as: behavioral variant frontotemporal degeneration (bvFTD), nonfluent/agrammatic primary progressive aphasias (naPPA), semantic variant PPA (svPPA), corticobasal syndrome (CBS), progressive supranuclear palsy (PSP), and/or frontotemporal degeneration with amyotrophic lateral sclerosis (FTD-ALS). There are associations between clinical phenotype and underlying pathology (3). Both svPPA (1, 4, 5) and FTD-ALS (1, 6) are most often associated with FTLD-TDP when sporadic (sFTLD-TDP). Other clinical syndromes are typically associated with FTLD-tau when sporadic (sFTLD-tau), such as PSP (7, 8), as well as naPPA (1, 4, 9) and CBS (1, 10, 11) once Alzheimer’s Disease has been ruled out (12). bvFTD can be associated with either sFTLD-tau or sFTLD-TDP with similar frequency (1, 13, 14). While pathology performed at autopsy remains the gold standard for diagnosis of FTLD, the ability to identify the differences between sFTLD-tau and sFTLD-TDP through *in vivo* imaging may be useful in monitoring protein-targeted clinical trials.

Arterial Spin Labeling (ASL) MRI non-invasively measures perfusion of brain tissue, quantified as cerebral blood flow (CBF), on a voxel-by-voxel basis. Although molecular imaging tracers for FTLD-tau and FTLD-TDP may more definitively detect pathological substrates associated with neurodegenerative diseases, ASL is safe and repeatable, does not use ionizing radiation (15), is likely to be useful for disease monitoring and progression, and can provide insight on how brain function is responding to therapeutic efforts (16). Comparisons of CBF derived from ASL can detect regions of hyper-or hypo-perfusion that may be early markers of neurodegenerative disease, like FTLD (17, 18). In the same imaging sessions as the ASL image, a structural T1-weighted (T1w) image may also be collected that can provide complementary information beneficial for quantifying and localizing CBF changes.

To facilitate accurate regional CBF calculations, the ASL image is aligned, or registered, to the T1w image; however, there exist obstacles in obtaining a perfect registration. For example, geometric distortions, caused by differences in acquisition parameters for ASL and T1w images, can complicate image registration (19). Also, ASL and T1w images have different spatial resolutions resulting in different voxel sizes; these different voxel sizes introduce difficulties for registration as the same voxel may contain signal from different amounts of grey matter (GM), white matter (WM), and cerebral spinal fluid, leading to partial volume effects (19). Furthermore, ASL images tend to have a low signal-to-noise ratio as labeled blood flow constitutes just 0.5–1.5% of the full tissue signal, resulting in images with noise that can make registration difficult. Additionally, as in any MRI modality, patient movement during image acquisition can introduce motion artifacts that compromise image quality, and lower image quality can also adversely affect image registration (18). To achieve accurate ASL-T1w alignment, registration methods should correct for spatial resolution differences, geometric distortion, partial volume effects, and motion artifacts (20). More accurate registrations should enable more sensitive detection of altered perfusion patterns due to increased accuracy of CBF quantification, which is useful in identifying regions of hypoperfusion in the brain (21). Determining best methods for accurate and automated ASL-T1w registration is imperative to better determine neuroanatomical patterns of hypoperfusion and to abate existing obstacles in interpretation across studies.

We hypothesize that we will find regional differences in perfusion in groups of patients with sFTLD-tau and sFTLD-TDP, with more predominant temporal perfusion in sFTLD-TDP and a more frontal distribution of hypoperfusion in sFTLD-tau. We also hypothesize that hypoperfusion will be associated with a general measure of cognitive and functional performance, the FTLD-modified clinical dementia rating scale sum-of-boxes (FTLD-CDR). Additionally, we analyze the performance of three ASL-T1w registration methods in patients with likely sFTLD pathology and cognitively normal control participants to better understand the accuracy and potential future clinical applicability of each registration approach. The three methods analyzed were a manual registration, functioning as a benchmark comparison, the manual registration as an “initialization” for Boundary-Based Registration (BBR) (22), and FMRIB’s Linear Imaging Registration Tool (FLIRT) (23) initialization followed by BBR. The Pearson’s Correlation Coefficient can be used to measure similarity between registered images by comparing vertex-wise mean CBF measurements (20). Since registrations supplemented by the non-rigid BBR transform will have the most degrees of freedom, we hypothesize that groups of images registered with BBR initialized using FLIRT (FLIRT+BBR) and with BBR initialized using manual (manual+BBR) will each be more similar on average compared to the manual registration, indicating better registration. We also hypothesize that FLIRT+BBR and manual+BBR will be more accurate, leading to increased sensitivity to subtle differences in the anatomical distribution of disease between patients with likely sFTLD-tau and likely sFTLD-TDP, relative to the manual registration alone.

## 2 Materials and methods

### 2.1 Participants

All data was collected through the Connectomic Imaging in Familial and Sporadic Frontotemporal Degeneration (FTDHCP). Participants were recruited at one of five centers in the United States: the University of California in San Francisco, Northwestern University, Mayo Clinic in Rochester, Massachusetts General Hospital, and the University of Pennsylvania. Before FTDHCP participant data collection began, each site was required to satisfy scanner hardware and software requirements, with all sites using a 3T Siemens Prisma scanner running VE11 and using a 64-channel head coil. Furthermore, each site was supplied with the same set of acquisition pulse sequences. Preliminary data was collected at each site and assessed to ensure adequate data quality was achieved before participant enrollment.

**Figure 1:**
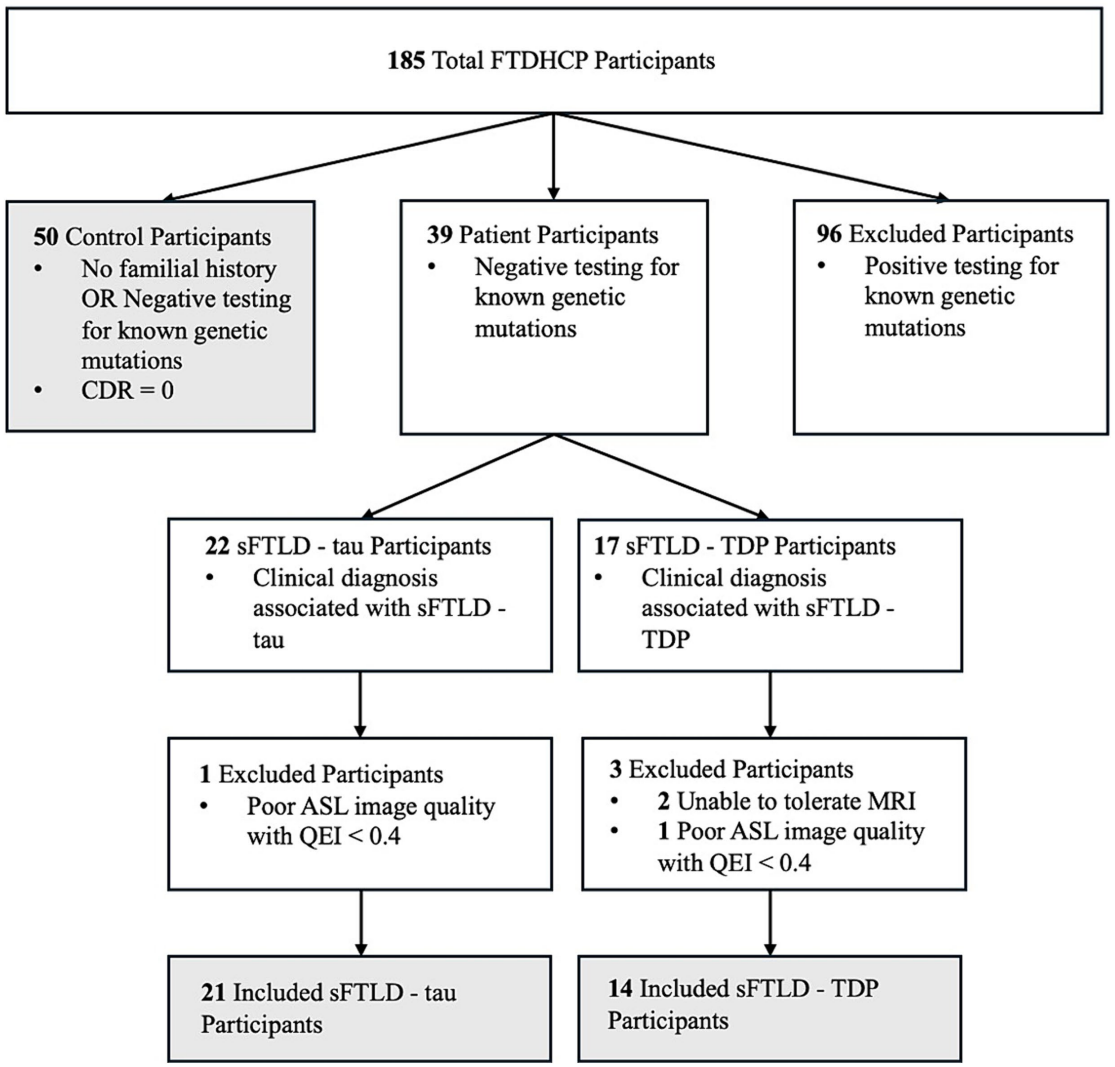
FTDHCP participant inclusion flowchart.

Three groups of participants were used for this study. The first was normal controls defined as participants who were cognitively normal (CDR = 0). The second group of participants were patients whose clinical diagnosis indicated likely sFTLD-tau due to a clinical diagnosis of PSP, naPPA, or CBS when Alzheimer’s Disease (AD) had been ruled out by cerebrospinal fluid beta-amyloid (1–42) level < 192 pg/mL or negative amyloid PET scan. The third group of participants were patients whose clinical diagnosis indicated likely sFTLD-TDP due to a clinical diagnosis of svPPA (with or without a co-diagnosis of bvFTD) or FTD-ALS. All patient participants additionally tested negative for mutations known to be associated with FTLD and are thus likely sFTLD. A schematic breakdown of FTDHCP participant inclusion criteria for the current study can be seen in Figure 1. All participants in the study completed an informed consent procedure approved by the IRB at the institution performing the data acquisition. All FTDHCP imaging data will be available through NIMH Data Archive (see Supplementary material). Participant demographics can be viewed in Table 1, and we compared groups of participants with sFTLD-tau and sFTLD-TDP using *t*-tests (ordinal variables) and chi-squared tests (categorical variables).

**Table 1:** Participant demographics.

Demographic characteristics	Control *N* = 50	sFTLD-TDP *N* = 14	sFTLD-tau *N* = 21
Male (*N*)	19	7	11
Female (*N*)	31	7	10
**Race (** *N* **)**			
White	47	13	17
Black or African American	2	1	2
Asian	1	0	1
Unknown	0	0	1
Education ± SD (years)	16.4 ± 2.40	15.9 ± 3.15	14.8 ± 2.62
Age at MRI ± SD (years)	49.3 ± 16.24	63.7 ± 5.38	67.5 ± 6.79
Age at Onset ± SD (years)	NA	59.8 ± 4.60	63.1 ± 7.07
FTLD-CDR ± SD	0 ± 0	6.82 ± 2.25	6.05 ± 3.51
**Phenotypic primary diagnosis (** *N* **)**			
naPPA	0	0	10
CBS	0	0	5
PSP	0	0	6
svPPA	0	11	0
bvFTD +svPPA	0	2	0
FTD-ALS	0	1	0

Summary of participant demographic statistics with associated standard deviations (SD). sFTLD-TDP, sporadic likely frontotemporal dementia due to TDP proteinopathy; sFTLD-tau, sporadic likely frontotemporal dementia due to tau proteinopathy; FTLD-CDR, Frontotemporal Lobar Degeneration modified Clinical Dementia Rating; naPPA, nonfluent/agrammatic variant primary progressive aphasia; svPPA, semantic variant primary progressive aphasia; CBS, corticobasal syndrome; PSP, progressive supranuclear palsy; bvFTD, behavioral variant frontotemporal dementia; FTD-ALS, frontotemporal dementia amyotrophic lateral sclerosis.

Additional inclusion criteria for this study included T1w and ASL images of adequate quality for analysis. Image quality for ASL was assessed quantitatively by mean CBF using the quality evaluation index (QEI) designed for ASL images (24, 25), with a QEI > 0.4 used as the criterion for adequate image quality as there was a break in the distribution of QEI values at this point, and the two below the break were confirmed to be of relatively poor quality by visual inspection (*N* = 2 excluded). Visual inspection of ASL images was also performed to assure no other obvious data quality issues in remaining images. T1w images were also assessed visually but were all deemed of adequate quality for analysis.

### 2.2 Image acquisition and ASLPrep processing

For ASL image acquisitions at all sites, a background-suppressed (BS) pseudo-continuous ASL (pCASL) pulse sequence with a 3D RARE stack-of-spirals readout and two-shot acceleration was used. The sequence acquisition parameters were TR = 4.2 s and TE = 10.03 ms, a voxel size of 3.75 $mm^{3}$ isotropic, no slice gap, a matrix of 64×64, and a flip angle of 90°. There was a total of 34 slices with a labeling duration of 1.8 s and a post labeling delay of 1.8 s. The labeling efficiency was 0.72. There were 14 label-control pairs to make 28 total volumes. An M0 (unweighted) image was acquired separately and used for quantification. We used one-way ANOVAS to ensure that image quality, as estimated using QEI, and mean gray matter CBF were not significantly different across sites [*F*(4) = 2.43, *p* = 0.054; *F*(4) = 1.33, *p* = 0.27, respectively].

*ASLPrep* v0.2.8 was used for ASL and T1w data preprocessing up to the point of ASL image registration to the T1w image (24). Many internal operations of *ASLPrep* are Python-based, using the *Nilearn* 0.8.1, *NumPy*, and *SciPy* packages. Briefly, ASL image preprocessing begins with a brain mask calculated for the ASL image. Head motion during image acquisition is corrected for with FSL mcflirt using rigid, 6 degree of freedom transforms. CBF is then computed at each voxel using a general kinetic model. The mean CBF is determined by averaging the CBF time series.

Anatomical T1w images were processed with *sMRIPrep* 0.6.1 as a part of ASLPrep. N4BiasFieldCorrection was used to correct for intensity non-uniformity which was followed by the *Nipype* implementation of antsBrainExtraction.sh with the OASIS30ANTs target template for skullstripping. FSL’s FAST was used for tissue segmentation of cerebrospinal fluid (CSF), white matter (WM), and gray matter (GM). Using antsRegistration (26), the T1w reference image was nonlinearly registered to the *ICBM 152 Nonlinear Asymmetrical template version 2009c* template.

The mean CBF and T1w images are aligned through different registration methods (see below) after preprocessing is completed. ASLPrep generates summary quality control HTML and csv files for each scan session, which were reviewed to ensure data quality as described above.

A high resolution T1w MPR image was also acquired in each session, with the following parameters TR = 2.4 s, TE = 2.22 ms, slice thickness = 0.8 mm, in plane resolution = 0.8 mm x 0.8 mm, a flip angle of 8°. Images were processed using Freesurfer’s recon-all v7.1.1.

### 2.3 Registration methods

For each participant, we considered three registration methods to align the mean CBF image and the processed T1w image acquired during the same scanning session. First, a manual registration was performed (“manual”) using Tkregister2. All manual registrations were performed by the same person and each registration was examined twice, in each case the transform was considered completed once the CBF image was overlaid as accurately as possible on the T1w image. The manual registration was considered a benchmark of comparison in this study. A FLIRT registration of the CBF image to the T1w images using 6 degrees of freedom (translational and rotational movement in all directions) was separately performed (23). The FLIRT and manual registrations were then each used as the initialization registration for BBR between the CBF and T1w images (22). Thus, “FLIRT+BBR” and a “manual+BBR” transforms were each generated. BBR is a non-rigid transform that, in addition to a preliminary registration with 6 degrees of freedom, also integrates a cost function along the vertices of a white and gray matter (GM-WM) cortical boundary of the T1w image. BBR spatially transforms this boundary to best align with the highest intensity gradients along the GM-WM boundary of the mean CBF image. Thus, BBR relies on a well-defined GM-WM boundary in the T1w image and a sufficient intensity gradient along the GM-WM boundary in the ASL image (22).

By default, FLIRT uses ASLPrep for ASL-T1w registration because it is considered both fast and accurate for multimodal registration (23). Thus, it was selected for this study as comparison to manual registration. For this study, both the manual and FLIRT registrations are rigid transformations using 6 degrees of freedom. BBR was chosen because it is a non-rigid transform that leverages a cost function to best align intensity gradients, importantly in our context at the GM-WM boundary (22).

By default, ASLPrep v0.2.8 uses a single ASL volume for registration to the T1w. The stack-of-spirals pCASL pulse sequence contains some “ringing” artifacts in individual volumes of the sequence, resulting in image intensity gradients that make registration to T1w difficult, regardless of the tools used, with automated methods failing for 10–50% of automated ASL-T1w registrations. However, the control-label subtraction and CBF quantification removes the artifact, so we used the mean CBF image as the moving image for alignment to the T1w image for all registration methods. This change resulted in all images of acceptable quality, as determined by a QEI > 0.4, to have adequate registrations, failing in just 2.2% (*N* = 2) of participants. These two participants were removed from all ensuing analysis to avoid biasing results. The code used to perform the automated CBF-T1w registrations is available publicly (https://github.com/ftdc-picsl/hcpASLregInTauTDP).

### 2.4 Registration comparisons

We projected the 3D volumetric CBF image to the cortical surface using each of the three transformation matrices generated using FreeSurfer (27). To compare registration methods, we used vertex-wise correlation coefficients as follows. A Pearson Correlation Coefficient (CC) was calculated for each pair of control mean CBF images in template space and separately for each pair of mean CBF images in template space for patients with sFTLD-tau and patients with sFTLD-TDP, resulting in 1225 unique pairwise comparisons in controls ( $\overset{50-1}{\underset{n=1}{\sum }}n=1225$ ), 210 unique pairwise comparisons in patients with sFTLD-tau ( $\overset{21-1}{\underset{n=1}{\sum }}n=210$ ), and 91 unique pairwise comparisons in patients with sFTLD-TDP ( $\overset{14-1}{\underset{n=1}{\sum }}n=91)$ . For the combined patient group CC values, the sFTLD-tau and sFTLD-TDP group CC values were combined, for a total of 301 unique pairwise comparisons. A CC value of 0 would represent two images with no linear relationship while a value of 1 would represent two identical images. For each of the control, all patients, sFTLD-tau, and sFTLD-TDP groups, CC values were compared with paired *t*-tests for each pair of registration methods, manual to manual+BBR, manual to FLIRT+BBR, and manual+BBR to FLIRT+BBR, to determine if registration methods were significantly different at a threshold *p* < 0.05, uncorrected for multiple comparisons.

Next, three mean CC values were calculated for each participant in the study, one for each registration method. Each mean participant CC value was calculated as the sum of CC values generated from pairwise comparisons divided by the total number of pairwise comparisons for the participant. To better understand differences in registration consistency between participant groups, *t*-tests of mean participant CC values between controls and all patients, controls and sFTLD-tau, controls and sFTLD-TDP, and participants with sFTLD-tau and sFTLD-TDP were determined for each registration method with a statistical significance threshold of *p* < 0.05 (uncorrected).

### 2.5 Mean CBF in regions of interest (ROIs)

The 219 cortical labels from the Lausanne125 parcellation (28) were normalized from template space to native T1w space for each participant, and mean CBF values for each participant were calculated in each ROI for each registration method. For each registration method, two-tailed two-sample *t*-tests of mean CBF were then performed in each ROI to determine regions of hypoperfusion in patients with sFTLD-tau relative to controls and patients with sFTLD-TDP relative to controls. *p*-values were adjusted for multiple comparisons using Bonferroni correction with a significance level of p_FWE_ < 0.05. Additionally, two-tailed two-sample *t*-tests of mean CBF were performed in each ROI to determine hypoperfusion in patients with sFTLD-TDP relative to sFTLD-tau with a significance level of p_FWE_ < 0.05 (Bonferroni-corrected).

### 2.6 FTLD-CDR statistical analysis

For sFTLD-tau and sFTLD-TDP participants, we used linear regression to relate regional CBF from each ROI in the Lausanne125 parcellation of the FLIRT+BBR registrations to the FTLD-CDR (29). The FTLD-CDR is a tool to measure cognitive and functional performance in patients with neurodegeneration, and in particular across the FTLD spectrum of disorders. As no results reached significance at the conservative threshold of p_FWE_ < 0.05, we report results at *p* < 0.05 (uncorrected). Two participants with sFTLD-tau were excluded from this analysis as they did not have available FTLD-CDR data.

### 2.7 Atrophy in ROIs

Again using the Lausanne125 parcellation, volumetric data for each participant was calculated in each ROI for the FLIRT+BBR registration. As with the mean CBF comparisons, two-tailed two-sample *t*-tests were performed in each ROI to detect atrophy in patients with sFTLD-tau relative to controls, patients with sFTLD-TDP relative to controls, and patients with sFTLD-TDP relative to sFTLD-tau. *p*-values were adjusted for multiple comparisons using Bonferroni correction with a significance level of p_FWE_ < 0.05. All statistics were performed in R.

## 3 Results

### 3.1 Participants

Patients with sFTLD-tau and sFTLD-TDP were not significantly different in age at MRI [*t*(33) = 1.84, *p* = 0.07], age at disease onset [*t*(33) = 1.95, *p* = 0.06], years of education [*t*(33) = −1.09, *p* = 0.28], sex [X^2^ (2, *N* = 1) = 0.02, *p* = 0.89], or, importantly, FTLD-CDR [*t*(33) = −0.77, *p* = 0.45].

### 3.2 Hypoperfusion in sFTLD-tau and sFTLD-TDP

To determine regions where perfusion differed between groups of patients and controls, *t*-tests of mean CBF in patients with sFTLD-tau and controls were performed in each ROI for each registration method. *T*-tests were similarly performed to compare patients with sFTLD-TDP and controls, and patients with likely sFTLD-tau to sFTLD-TDP. All regions of hypoperfusion were determined significant at p_FWE_ < 0.05 (Bonferroni-corrected). As *t*-tests were two-tailed, our tests were sensitive to both hypo-and hyperperfusion, yet we only detected hypoperfusion in all comparisons.

**Figure 2:**
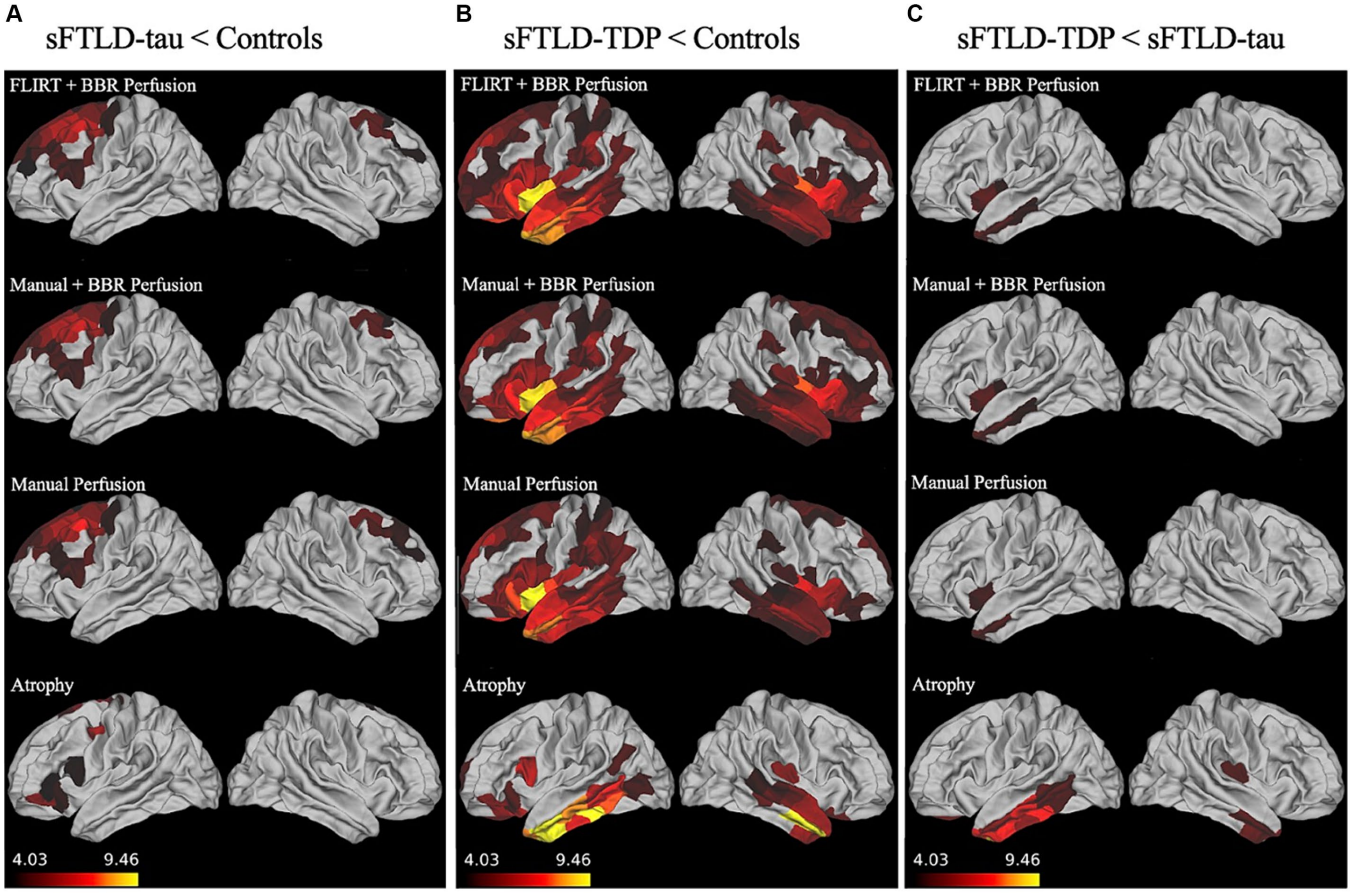
Whole-brain perfusion comparisons. **(A)** Hypoperfusion and atrophy patterns in sporadic likely frontotemporal lobar degeneration due to tau (sFTLD-tau) relative to controls. **(B)** Hypoperfusion and atrophy patterns in sporadic likely frontotemporal lobar degeneration due to TDP (sFTLD-TDP) relative to controls. **(C)** Hypoperfusion and atrophy patterns in sFTLD-TDP relative to sFTLD-tau. All results shown are considered significant at *p* < 0.05 after Bonferroni correction for multiple comparisons. Regions of more significant hypoperfusion (larger *t*-statistics) are shown in lighter colors (yellow), and all brain mappings use the same scale.

As can be seen in Figure 2A, in patients with sFTLD-tau relative to controls, all three registration methods detected hypoperfusion in the bilateral middle and superior frontal gyrus, left precentral gyrus, and left inferior frontal gyrus.

As shown in Figure 2B, patients with sFTLD-TDP show more hypoperfusion relative to controls than sFTLD-tau, with hypoperfusion in bilateral insular cortex, occipitotemporal gyrus, anterior cingulate cortex, orbitofrontal cortex, temporal pole, superior, inferior, and middle temporal gyrus, limbic regions, precentral gyrus, superior and inferior middle frontal gyri, in addition to left middle frontal and cingulate gyri, and right parahippocampal gyrus. Additionally, FLIRT+BBR also detected hypoperfusion in the left superior temporal sulcus and postcentral gyrus, bilateral regions of parietal lobe, and paracentral lobules, as well as right cingulate gyrus and Heschl’s gyrus. When comparing regions following manual registration, hypoperfusion was also detected in the right cingulate gyrus and parietal cortex, left frontal pole, temporal sulcus, and parahippocampal gyrus, and bilateral regions of occipital lobe, paracentral gyrus, and supramarginal gyrus. Finally, when using manual+BBR, hypoperfusion can also be seen in right middle frontal, parahippocampal, and Heschl’s gyri, along with left parietal cortex, and bilateral paracentral lobule and regions of occipital lobe.

As can be seen in Figure 2C, all three registration methods detected hypoperfusion in sFTLD-TDP relative to sFTLD-tau in the left insular cortex, left middle temporal gyrus, and the left temporal pole when comparing sFTLD-TDP relative to sFTLD-tau. No hypoperfusion was found in sFTLD-tau relative to sFTLD-TDP.

A complete list of identified regions for each registration method for each comparison can be found in the Supplementary materials.

### 3.3 Relationship between perfusion and FTLD-CDR

**Figure 3:**
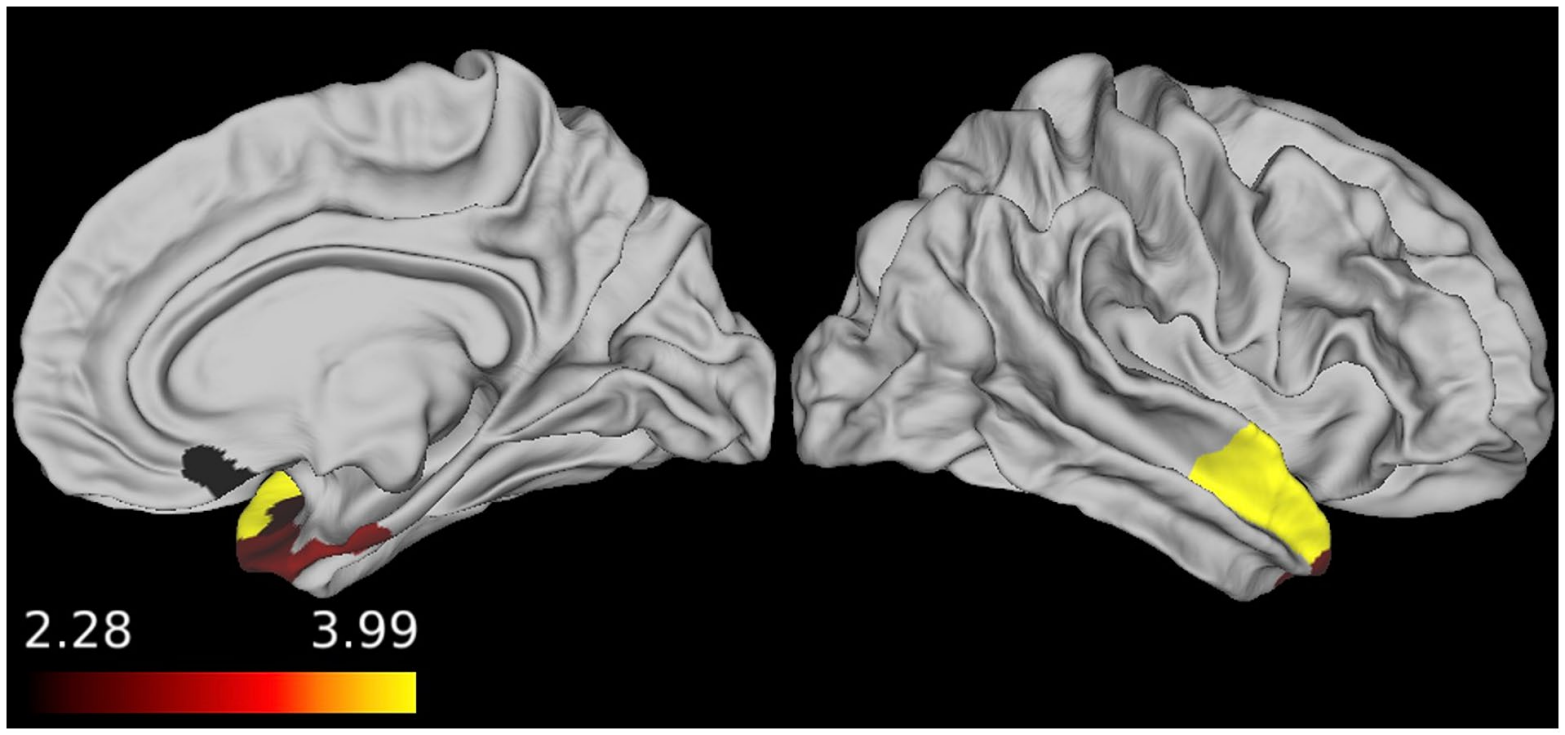
Regional associations between FTLD-CDR and cerebral blood flow (CBF). In patients with sporadic likely frontotemporal lobar degeneration due to TDP (sFTLD-TDP), the FTLD clinical dementia rating sum-of-boxes score was inversely associated with CBF, demonstrating that as perfusion decreases, disease severity increases (*p* < 0.05, uncorrected).

To determine whether there was an association between perfusion and disease severity in participants with sFTLD-TDP and sFTLD-tau, linear regressions were conducted in each patient group relating mean CBF in each ROI to FTLD-CDR. As shown in Figure 3, for participants with sFTLD-TDP, the right medial orbitofrontal cortex, right entorhinal cortex, right temporal pole, and right superior temporal gyrus are inversely associated with FTLD-CDR (*p* < 0.05). In participants with sFTLD-tau, no regions were significantly associated with FTLD-CDR (all *p* > 0.05).

### 3.4 Atrophy in sFTLD-tau and sFTLD-TDP

We examined patterns of atrophy in sFTLD-tau relative to controls, sFTLD-TDP relative to controls, and between the groups of patients with sFTLD-TDP and sFTLD-tau. As seen in Figure 2A, patients with sFTLD-tau show atrophy relative to controls in the left orbitofrontal, inferior and superior frontal regions, as well as left precentral gyrus and right superior frontal gyrus. In Figure 2B, it can be seen that sFTLD-TDP shows extensive atrophy in bilateral temporal lobes and the orbitofrontal cortex. Additionally, atrophy is detected in the left cingulate cortex, inferior frontal gyrus, prefrontal cortex, and right insula. As seen in Figure 2C, patients with sFTLD-TDP show atrophy relative to sFTLD-tau in the bilateral inferior and middle temporal gyrus, medial temporal lobe, and orbitofrontal cortex, as well as the right insula.

### 3.5 Statistical comparison of registration methods

Paired *t*-tests of participant CC values were conducted to estimate differences between registration methods within control participants, within all patients, and within participants with sFTLD-tau or sFTLD-TDP (see summary, Table 2). Among controls, all registration methods were significantly different (*p* < 0.001) from each other with the greatest *t*-statistic differences noted between registrations supplemented with BBR (FLIRT+BBR and manual+BBR) and the manual registration [*t*(2448) = 50.8 and *t*(2448) = 48.5, respectively]. The smallest *t*-statistic can be noted between FLIRT+BBR and manual+BBR [*t*(2448) = 6.33]. Among patient participants, the FLIRT+BBR and manual+BBR registrations were not significantly different [*t*(600) = −0.01, *p* = 0.991], yet both these registration methods produced significantly different results when compared to the manual registration [*t*(600) = 19.6, *p* < 0.001 and *t*(600) = 19.8, *p* < 0.001, respectively]. It can be noted that the *t*-statistic values were similar when comparing FLIRT+BBR and manual+BBR to the manual registration in patient participants. However, when comparing registrations within patient groups, FLIRT+BBR vs. manual+BBR were significantly different from each other in both sFTLD-tau and sFTLD-TDP [*t*(418) = 2.2, *p* < 0.05 and *t*(180) = −2.2, *p* < 0.05, respectively] (Table 2).

**Table 2:** Statistical comparison of registration methods.

Control participants								
FLIRT + BBR *vs* Manual + BBR			FLIRT + BBR *vs* Manual			Manual + BBR *vs* Manual		
*p*	*t*	Cohen’s d	*p*	*t*	Cohen’s d	*p*	*t*	Cohen’s d
<0.001	6.334	0.009	<0.001	50.811	0.338	<0.001	48.462	0.329
**All patient participants**								
FLIRT + BBR *vs* Manual + BBR			FLIRT + BBR *vs* Manual			Manual + BBR *vs* Manual		
*p*	*t*	Cohen’s d	*p*	*t*	Cohen’s d	*p*	*t*	Cohen’s d
0.991	−0.011	<−0.001	<0.001	19.621	0.234	<0.001	19.782	0.234
**sFTLD – tau Participants**								
FLIRT + BBR *vs* Manual + BBR			FLIRT + BBR *vs* Manual			Manual + BBR *vs* Manual		
*p*	*t*	Cohen’s d	*p*	*t*	Cohen’s d	*p*	*t*	Cohen’s d
0.027	2.221	0.004	<0.001	18.098	0.306	<0.001	17.629	0.302
**sFTLD – TDP Participants**								
FLIRT + BBR *vs* Manual + BBR			FLIRT + BBR *vs* Manual			Manual + BBR *vs* Manual		
*p*	*t*	Cohen’s d	*p*	*t*	Cohen’s d	*p*	*t*	Cohen’s d
0.0162	−2.451	−0.006	<0.001	8.625	0.155	<0.001	9.351	0.161

Paired *t*-tests and effect sizes (Cohen’s d) of Pearson’s Correlation Coefficient (CC) values generated from unique pairwise comparisons in participants between each registration method were determined for both control and patient groups. Patient participants were classified as either sporadic likely frontotemporal lobar degeneration due to tau (sFTLD-tau) or sporadic likely frontotemporal lobar degeneration due to TDP (sFTLD-TDP). BBR, Boundary-Based Registration; FLIRT, FMRIB’s Linear Registration Tool; *p*, *p*-value; *t*, *t*-statistic.

**Figure 4:**
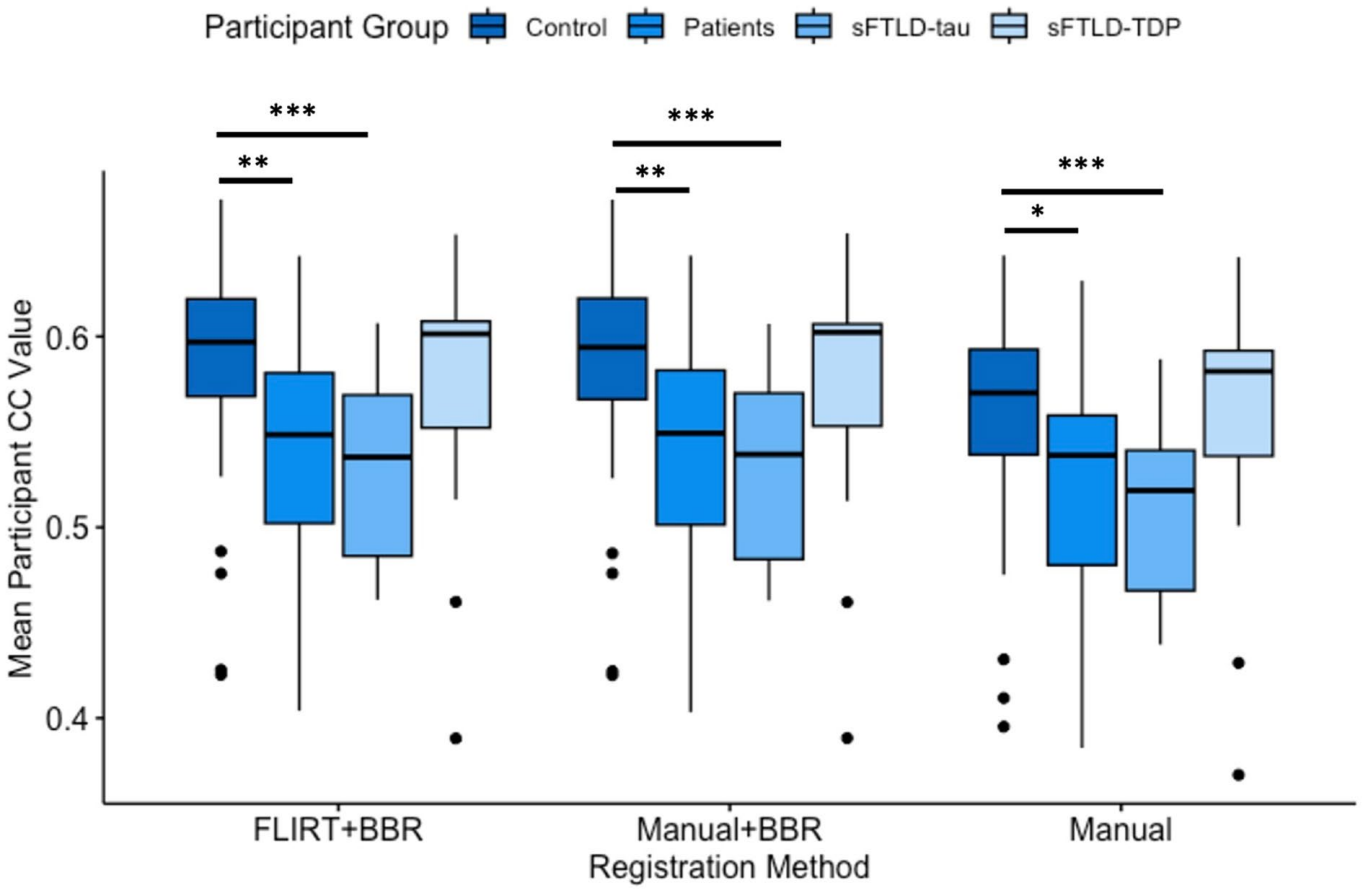
Comparison of mean participant Pearson’s Correlation Coefficient (CC) values. Comparisons between controls and all patients were significantly different in all registration methods (* represents *p* < 0.05; ** represents *p* < 0.01). Comparisons between controls and sporadic likely frontotemporal lobar degeneration due to tau (sFTLD-tau) were significantly different in all registration methods (*p* < 0.001, denoted ***). Controls relative to sporadic likely frontotemporal lobar degeneration due to TDP (sFTLD-TDP) and sFTLD-tau relative to sFTLD-TDP were not significantly different (*p* > 0.05). BBR, Boundary-Based Registration; FLIRT, FMRIB’s Linear Registration Tool.

Average mean participant CC values were determined for each patient group and all registration methods. As greater CC values indicate more consistent registrations, both registrations supplemented by BBR performed better on average in all participant groups than solely the manual registration, as shown in Figure 4. Additionally, these findings suggest BBR performed similarly regardless of being initialized with either the FLIRT registration or the manual registration in all participant groups (Table 3; Figure 4).

**Table 3:** Comparison of average mean participant CC value between registration methods.

	Controls	All Patients	sFTLD-tau	sFTLD-TDP
FLIRT + BBR	0.5866 ± 0.0521	0.5485 ± 0.0589	0.5341 ± 0.0454	0.5702 ± 0.0712
Manual + BBR	0.5859 ± 0.0521	0.5486 ± 0.0594	0.5338 ± 0.0459	0.5709 ± 0.0715
Manual	0.5603 ± 0.0519	0.5291 ± 0.0612	0.5130 ± 0.0461	0.5532 ± 0.0742

Average of mean participant CC values and associated standard deviation for all participants in each group classification were determined for each registration method. Larger CC values indicate more consistent registrations across participants. *T*-tests were conducted between controls, all patients, sFTLD-tau, and sFTLD-TDP. Patient participants were classified as sporadic likely frontotemporal lobar degeneration due to tau (sFTLD-tau) or sporadic likely frontotemporal lobar degeneration due to TDP (sFTLD-TDP). BBR, Boundary-Based Registration; FLIRT, FMRIB’s Linear Registration Tool; CC, Pearson’s Correlation Coefficient.

We used *t*-tests to compare mean participant CC values between controls and sFTLD-tau, between controls and sFTLD-TDP, between controls and all patients, and between sFTLD-tau and sFTLD-TDP for each registration method. As shown in Figure 4 and Table 3, control participants demonstrated greater CCs than participants with sFTLD-tau (*p* < 0.001). There were no significant differences in mean participant CC values between control participants and participants with sFTLD-TDP [*t*(62) = 0.805, *p* = 0.43 for FLIRT+BBR, *t*(62) = 0.74, *p* = 0.47 for manual+BBR, and *t*(62) = 0.34, *p* = 0.74 for manual registrations] or between participants with sFTLD-tau and sFTLD-TDP [*t*(33) = −1.68, *p* = 0.11 for FLIRT+BBR, *t*(33) = −1.72, *p* = 0.10 for manual+BBR, and *t*(33) = −1.81, *p* = 0.09 for manual registrations]. In all three registration methods, mean participant CC values between controls and the combined patient participants group were significantly different [*t*(83) = 3.07, *p* < 0.01 for FLIRT+BBR, *t*(83) = 2.99, *p* < 0.01 for manual+BBR, *t*(83) = 2.46, *p* < 0.05 for manual].

Additionally, the mean time for the FLIRT+BBR registration was 897 s (±167 s). For the manual+BBR registrations, the mean time to complete the BBR portion was 846 s (± 118 s). In both cases, this includes the amount of time to perform the necessary file type conversions to create all files for BBR. It is also worth noting that with the available computational resources, 16 registrations could be performed simultaneously by one user. The precise mean time for each manual registration was not available, but author SM, who performed each manual registration, estimates it took approximately 1800 s per image on average.

## 4 Discussion

In this study, we compared regional CBF between groups of patients with likely sFTLD-tau and likely sFTLD-TDP, finding unique regions of hypoperfusion for each group relative to controls. Importantly, we were also able to detect distinct regions of hypoperfusion in sFTLD-TDP relative to sFTLD-tau. Furthermore, we related hypoperfusion in sFTLD-TDP to disease severity, adding evidence that detected hypoperfusion is relevant clinically. We also evaluated FLIRT+BBR, manual+BBR, and manual methods for registering CBF images to T1w images, finding similar results in each case at the group level and providing evidence that the automated FLIRT+BBR is ideal for registration.

### 4.1 Hypoperfusion patterns in sFTLD-tau and sFTLD-TDP

We found that patients with likely sFTLD-tau demonstrated hypoperfusion in bilateral middle frontal gyrus, bilateral superior frontal gyrus, left precentral gyrus, and left inferior frontal gyrus relative to controls. Past work identified that patients with CBS demonstrated hypoperfusion in the left inferior frontal gyrus, left parahippocampal gyrus, right superior frontal gyrus, right insula, and right cuneus relative to controls (30). Additionally, other studies combining patients with PSP and CBS found regional hypoperfusion in the thalamus, caudate nucleus, anterior cingulate cortex, superior and frontal gyri, and temporal lobe (31). A study highlighting regions of hypoperfusion in neurodegenerative diseases associated with FTLD-tau identified the prefrontal cortex, dorsolateral frontal cortex, orbitofrontal cortex, anterior cingulate cortex (32). Furthermore, a study reviewing primary progressive aphasias noted patients with naPPA have hypometabolism using FDG-PET, a close proxy to hypoperfusion using ASL MRI, in the inferior frontal gyrus, superior temporal gyrus, and inferior parietal lobule (33). Some differences in regional hypoperfusion between studies can be attributed to the classification of participant data pools. This study classified sFTLD-tau as patients with CBS, naPPA, and PSP, and though similar regions are detected, this study does not cover the full breadth of regions identified in other studies likely due to the differences in the proportions of participants with the phenotypes considered. Many regions of atrophy were overlapping regions of hypoperfusion in sFTLD-tau relative to controls, while other regions of atrophy were adjacent to regions of hypoperfusion. Previous work examining both GM volumes and GM CBF together improve the predictive power of MRI for pathologic burden measured at autopsy (34), so our results may provide converging evidence that T1w and ASL are likely capturing some similar and some unique aspects of the neurodegenerative process in sFTLD-tau.

We found that patients with likely sFTLD-TDP demonstrated extensive hypoperfusion in bilateral frontal and temporal cortex, largely consistent with regions demonstrating early pathology in a prior autopsy study looking at bvFTD patients with FTLD-TDP (2). For sFTLD-TDP, the majority of participants in this study were patients with svPPA. Previous literature comparing regional hypoperfusion in patients with svPPA relative to controls using ASL-MRI detected very similar regions of hypoperfusion to those found in our study (17). Others have found smaller numbers of regions, like solely the left parietal associative cortex, posterior cingulate, anterior cingulate, and orbitofrontal cortex (35). Our results also display some similarities to patterns of hypoperfusion found in patients diagnosed with bvFTD, some of whom may also develop svPPA symptoms (13) relative to controls such as the bilateral orbitofrontal cortex, left dorsolateral prefrontal cortex, left middle temporal gyrus, left superior frontal cortex, and the insula (36). Again, differing regional hypoperfusion across studies may be explained by the differing proportions of patients with specific disease diagnosis in the patient populations. Interestingly, all regions of atrophy in sFTLD-TDP relative to controls also demonstrated hypoperfusion (for at least one of the registration methods), though with many regions of hypoperfusion not demonstrating atrophy. This may be due to ASL being sensitive to reduced neural function occurring before frank neurodegeneration. The more widespread hypoperfusion patterns shown here may be due, at least in part, to increased sensitivity to perfusion changes as a direct result of our carefully performed ASL-T1w registrations.

### 4.2 Associations between CBF and disease severity

In patients with sFTLD-TDP, we found that the FTLD-CDR was inversely associated with CBF in regions demonstrating hypoperfusion, namely the right medial orbitofrontal cortex, entorhinal cortex, temporal pole, and superior temporal gyrus. These findings support the idea that perfusion decreases as cognitive function declines in sFTLD-TDP, further demonstrating the validity of CBF as a measure of disease severity in this patient population. Interestingly, autopsy studies have previously shown these as regions with early pathology in bvFTD patients with FTLD-TDP (2). It is worth noting that the patient population in participants with sFTLD-TDP is predominantly svPPA, which is most often associated with atrophy in the left temporal lobe, as opposed to the right temporal lobe results found here. This may indicate that perfusion in the left hemisphere is already at “floor” so the disease severity measured with the FTLD-CDR is more sensitive to variance associated with functional decline in right temporal regions, which have been previously implicated in groups of patients with both svPPA and bvFTD with likely FTLD-TDP (17, 37). Taken together, our findings demonstrate the utility of ASL-MRI as a tool to detect hypoperfusion associated with disease in patients with FTLD.

For participants with sFTLD-tau, no ROIs were significantly associated with FTLD-CDR, again likely due to the more varied patient phenotypes present in this group compared to the homogeneity in the sFTLD-TDP group. Though CBF has previously been linked to pathologic burden in FTLD-tau (34), future work with adequately powered samples should investigate correlations between CBF and cognition to better understand the clinical relevance of ASL-MRI in participants with sFTLD-tau, as well as in different clinical phenotypes associated with sFTLD-tau.

### 4.3 Distinct hypoperfusion patterns in sFTLD-tau and sFTLD-TDP

Our findings indicate that ASL imaging may have value in discriminating between sFTLD-tau and sFTLD-TDP, and provide insight on differences in disease processes between them. Previous work has analyzed perfusion patterns in patients with FTLD relative to controls and relative to AD patients (38), yet it remains important to examine perfusion patterns between patients with likely sFTLD-tau and likely sFTLD-TDP. Previous literature comparing perfusion in familial FTLD-tau to FTLD-TDP identified the same regions of left inferior, middle, and superior temporal gyri, with the differing regions of the right inferior and medial gyri, right rectal gyrus, and left inferior occipital gyrus (39). These discrepancies may be due to the differing definitions of FTLD-tau and FTLD-TDP in the studies, as well as potential differences in sporadic vs. familial patterns of disease, even within the same pathology (9). In our study, sFTLD-tau and sFTLD-TDP groups were determined from clinical diagnosis, which is necessary to complete antemortem in the absence of genetic mutations, though this is also a limitation as using clinical phenotype to estimate underlying pathology is not perfect. Small differences may arise from regional disease patterns due to more widespread dystrophic microglia across cortical layers and greater problems in astrocytes surrounding vessels in FTLD-TDP relative to FTLD-tau (40). Phenotypic differences between the cohorts may also account for regional differences. Nevertheless, registration with all three methods revealed similar hypoperfusion patterns between sFTLD-tau relative to controls and sFTLD-TDP relative to controls. Additionally, all three registration methods found similar regional patterns of hypoperfusion in likely sFTLD-TDP relative to likely sFTLD-tau when directly comparing those groups. The different regions of hypoperfusion between sFTLD-tau and sFTLD-TDP can therefore be attributed to differences in disease, as opposed to variations in registration performance.

### 4.4 Image registration method comparison

When comparing detected regions of hypoperfusion across all three registration methods, all methods had a similar sensitivity in revealing regional hypoperfusion, with only subtle differences in affected ROIs and *t*-statistics. It is suspected that registration methods that are more effective in aligning the T1w and CBF image will yield a more accurate analysis of perfusion patterns, which has important implications for the potential clinical use of ASL (20, 22). Our results suggest that the automated FLIRT+BBR registration produces sufficient image alignments to accurately detect regional hypoperfusion, as demonstrated relative to the manual registration.

Our results demonstrate that using FLIRT+BBR to register CBF and T1w images is a consistent and accurate method to be used on both patient and control participants. Importantly, FLIRT+BBR can effectively replace time-intensive manual registration efforts allowing ASL-T1w image registrations to be completed automatically, accurately, and without user-specific bias (41, 42). Clinically, perfusion patterns derived from CBF of ASL-MRI imaging could be reliably performed using the fully automated FLIRT+BBR to better identify disease progression or to monitor protein-targeted clinical trials.

Additionally, results demonstrated that FLIRT+BBR and manual+BBR generated transformations that were similar to each other within groups of participants, and these methods resulted in more consistent registrations across participants than the manual registration. FLIRT and manual are rigid transforms while BBR is a non-rigid transform that, in addition to 6 degrees of freedom, also implements a cost function that regionally aligns WM/GM boundary gradients of the CBF and T1w images (22). This feature likely explains why the registrations supplemented by BBR performed similarly to each other, whether initialized by manual registration or FLIRT, and why BBR may generally perform more consistently than the manual registration.

We showed, by comparing mean participant CC values, that control participants were more consistently registered relative to participants with sFTLD-tau with no significant differences between controls and sFTLD-TDP and between sFTLD-tau and sFTLD-TDP. We also showed that control participants were more consistently registered relative to a combined group of all patients. The patient groups in this study are determined by likely pathology as estimated by clinical phenotypes, meaning that each patient pathology group is composed of multiple different neurodegenerative disease phenotypes. Participants in the sFTLD-tau patient group were composed of 3 different clinical phenotypes in more varied proportions (naPPA = 10, CBS = 5, PSP = 6) relative to participants with likely sFTLD-TDP who were mainly diagnosed with svPPA (svPPA = 11, bvFTD+svPPA = 2, FTD-ALS = 1). Past work examining patients with likely FTLD-TDP with either svPPA (17), ALS or bvFTD (36) found that groups of patients with these phenotypes have some similar, as well as some distinct regions of hypoperfusion. Comparisons of CC between controls and participants with sFTLD-TDP were not significantly different likely because participants in each group were, respectively, more homogenous; controls were composed of participants with no clinical neurodegeneration and sFTLD-TDP was composed of patient participants, the vast majority of whom were noted to have features of svPPA. As discussed above, patients with likely sFTLD-tau, those with naPPA, PSP, and CBS, are also likely to have some similar but also unique regions of hypoperfusion (43). Thus, it is likely that different clinical syndromes with the same proteinopathy will have different regional hypoperfusion as well as likely different atrophied regions (44). Within the context of the CC calculation, different regional hypoperfusion corresponding to different clinical phenotypes will result in lower CC, even if the registration is performing reasonably. This effect is notable in the likely sFTLD-tau participant group where there is the most variation in participant phenotypes, and to a lesser effect in the likely sFTLD-TDP group, likely explaining why no significant differences in registration consistency were detected between these two groups. Control participant brains are assumed to not be affected by disease-related processes that may alter regional perfusion patterns, such as reduced cortical functioning or cortical atrophy like the patient brains (38) despite known changes that occur during “normal” aging (45). CBF maps derived from control participant brain scans were more consistently registered relative to patients with sFTLD-tau scans, and this is likely because images that are already more similar will likely also be registered more similarly. On a similar note, control participants were shown to be better registered than the combined group of all patient participants likely due to the homogeneity in control participants relative to the diversity of regional perfusion patterns in a combined group of sFTLD-tau and sFTLD-TDP patients.

### 4.5 Limitations and future directions

The primary focus of this study was determining regions of hypoperfusion in patients with sFTLD-tau and sFTLD-TDP. Though we investigated measures of both GM structure and GM function, future work should also examine WM using diffusion-weighted imaging to better understand the complex structure–function relationships mediated by WM connections in the brains of patients with FTLD (46).

Patients were classified as either likely sFLTD-tau or likely sFTLD-TDP based on clinical phenotypes; however, these classifications were not pathologically confirmed. Yet, a strength of this work is that all patients were confirmed to not have any known mutations associated with FTLD, as there may be differences in mechanisms of disease between those with familial FTLD and those with sFTLD (9). Though we found relatively consistent group differences for all registration methods, we found subtle differences in regions detected, which may be attributable to our study combining a number of diagnoses in our likely sFTLD-tau and likely sFTLD-TDP groups. Future work should examine ASL-MRI images in adequately powered samples from patients that have been pathologically confirmed as sFLTD-tau or sFTLD-TDP to better determine the true distribution of functional changes in patients with sFTLD. Only hypoperfusion was detected in this work, yet investigating potential hyperperfusion patterns, especially in cases of asymptomatic familial FTLD, should be a focus in future work.

We postulated that increased CC values between participant CBF images in template space indicates better anatomical alignment in the intra-subject CBF to T1w registration. We did not evaluate the quality of the T1w to template registration, which may also vary between patient groups, and which may limit our ability to detect differences in CBF to T1w normalization (20). Even under optimal anatomical alignment, the CC value comparison would not be 1 because of genuine population variance and measurement error in the CBF itself. However, given that the inter-subject CBF similarity is never explicitly optimized in the pipeline, it is unlikely that the improvements in CC values from BBR are due to overfitting. Different ASL acquisition protocols may have different characteristic SNR and distortion, so results should be validated on datasets using other protocols (20).

### 4.6 Conclusion

We have shown that ASL is effective in detecting regions of hypoperfusion for patients with sFTLD relative to controls. Additionally, comparisons of CBF detected differences in regional perfusion patterns between groups of participants with likely sFTLD-tau and likely sFTLD-TDP. Furthermore, we showed that a measure of disease severity was related to hypoperfusion in patients with sFTLD-TDP, indicating that ASL is sensitive to clinically relevant changes in brain function. Our results demonstrate that FLIRT+BBR can register ASL and T1w images at least as well as manual registration. Additionally, we showed that using FLIRT+BBR registration produces consistent results in both control and patient participants. Different registration methods demonstrated subtle differences in regions detected in group comparisons; yet, the consistency of detected regions of hypoperfusion for all methods indicates that ASL-MRI can serve as a useful modality to determine regional hypoperfusion when examining patients with sFTLD.

## Data Availability

Publicly available datasets were analyzed in this study. This data can be found at: Code that was used to perform CBF-T1w registrations is available in a public repository (https://github.com/ftdc-picsl/hcpASLregInTauTDP). FTDHCP imaging data is available in a NIMH Data Archive (https://nda.nih.gov/edit_collection.html?id=3160).

## References

[1] Irwin DJ Cairns NJ Grossman M McMillan CT Lee EB Van Deerlin VM. Frontotemporal lobar degeneration: defining phenotypic diversity through personalized medicine. Acta Neuropathol. (2015) 129:469–91. doi: 10.1007/s00401-014-1380-1, PMID: 25549971 PMC4369168

[2] Brettschneider J Del Tredici K Irwin DJ Grossman M Robinson JL Toledo JB. Sequential distribution of pTDP-43 pathology in behavioral variant frontotemporal dementia (bv FTD). Acta Neuropathol. (2014) 127:423–39. doi: 10.1007/s00401-013-1238-y, PMID: 24407427 PMC3971993

[3] Grossman M. Primary progressive aphasia: Clinicopathological correlations. Nat Rev Neurol. (2010) 6:88–97. doi: 10.1038/nrneurol.2009.216, PMID: 20139998 PMC3637977

[4] Giannini LAA Xie SX McMillan CT Liang M Williams A Jester C. Divergent patterns of TDP-43 and tau pathologies in primary progressive aphasia. Ann Neurol. (2019) 85:630–43. doi: 10.1002/ana.25465, PMID: 30851133 PMC6538935

[5] Borghesani V Battistella G Mandelli ML Welch A Weis E Younes K. Regional and hemispheric susceptibility of the temporal lobe to FTLD-TDP type C pathology. Neuro Image. (2020) 28:102369. doi: 10.1016/j.nicl.2020.102369, PMID: 32798912 PMC7426562

[6] Tan RH Kril JJ Fatima M McGeachie A McCann H Shepherd C. TDP-43 proteinopathies: pathological identification of brain regions differentiating clinical phenotypes. Brain. (2015) 138:3110–22. doi: 10.1093/brain/awv220, PMID: 26231953

[7] Dickson DW Rademakers R Hutton ML. Progressive Supranuclear palsy: pathology and genetics. Brain Pathol. (2015) 17:74–82. doi: 10.1111/j.17503639.2007.00054.x PMC8095545 17493041

[8] Kovacs GG Lukic MJ Irwin DJ Arzberger T Respondek G Lee EB. Distribution patterns of tau pathology in progressive supranuclear palsy. Acta Neuropathol. (2020) 140:99–119. doi: 10.1007/s00401-020-02158-2, PMID: 32383020 PMC7360645

[9] Giannini LAA Peterson C Ohm D Xie SX McMillan CT Raskovsky K. Frontotemporal lobar degeneration proteinopathies have disparate microscopic patterns of white and grey matter pathology. Acta Neuropathol Commun. (2021) 9:30. doi: 10.1186/s40478-021-01129-2, PMID: 33622418 PMC7901087

[10] Kouri N Murray ME Hassan A Rademakers R Uitti RJ Boeve BF. Neuropathological features of corticobasal degeneration presenting as corticobasal syndrome or Richardson syndrome. Brain. (2011) 134:3264–75. doi: 10.1093/brain/awr234 21933807 PMC3212714

[11] Niccolini F Wilson H Hirschbichler S Yousaf T Pagano G Whittington A. Disease-related patterns of in vivo pathology in Corticobasal syndrome. Eur J Nucl Med Mol Imaging. (2018) 45:2413–25. doi: 10.1007/s00259-018-4104-2, PMID: 30090966 PMC6208819

[12] Hassan A Whitwell JL Josephs KA. The corticobasal syndrome–Alzheimer’s disease conundrum. Expert Rev Neurother. (2011) 11:1569–78. doi: 10.1586/ern.11.153, PMID: 22014136 PMC3232678

[13] Miki T Yokota O Ishizu H Kuroda S Oshima E Terada S. Behavioral variant of frontotemporal dementia: fundamental clinical issues associated with prediction of pathological bases. Neuropathology. (2016) 36:388–404. doi: 10.1111/neup.12290, PMID: 26969837

[14] Perry DC Brown JA Possin KL Datta S Trujillo A Radke A. Clinicopathological correlations in behavioural variant frontotemporal dementia. Brain. (2017) 140:3329–45. doi: 10.1093/brain/awx254, PMID: 29053860 PMC5841140

[15] Detre JA Leigh JS Williams DS Koretsky AP. Perfusion imaging. Magn Reson Med. (1992) 23:37–45. doi: 10.1002/mrm.1910230106 1734182

[16] Wolk DA Detre JA. Arterial spin labeling MRI: an emerging biomarker for Alzheimer’s disease and other neurodegenerative conditions. Curr Opin Neurol. (2012) 25:421–8. doi: 10.1097/WCO.0b013e328354ff0a, PMID: 22610458 PMC3642866

[17] Olm CA Kandel BM Avants BB Detre JA Gee JC Grossman M. Arterial spin labeling perfusion predicts longitudinal decline in semantic variant primary progressive aphasia. J Neurol. (2016) 263:1927–38. doi: 10.1007/s00415-016-8221-1, PMID: 27379517 PMC5097861

[18] Petcharunpaisan S Ramalho J Castillo M. Arterial spin labeling in neuroimaging. World J Radiol. (2010) 2:384–98. doi: 10.4329/wjr.v2.i10.384, PMID: 21161024 PMC2999014

[19] Petr J Mutsaerts HJMM De Vita E Steketee RME Smits M Nederveen AJ. Effects of systematic partial volume errors on the estimation of gray matter cerebral blood flow with arterial spin labeling MRI. MAGMA. (2018) 31:725–34. doi: 10.1007/s10334-018-0691-y, PMID: 29916058

[20] Mutsaerts HJ Petr J Thomas DL de Vita E Cash DM van Osch MJ. Comparison of arterial spin labeling registration strategies in the multi-Centre GENetic frontotemporal dementia initiative (GENFI). J Magn Reson Imaging. (2018) 47:131–40. doi: 10.1002/jmri.25751, PMID: 28480617 PMC6485386

[21] Wong EC. An introduction to ASL labeling techniques. J Magn Reson Imaging. (2014) 40:1–10. doi: 10.1002/jmri.24565 24424918

[22] Greve DN Fischl B. Accurate and robust brain image alignment using boundary-based registration. Neuro Image. (2009) 48:63–72. doi: 10.1016/j.neuroimage.2009.06.060, PMID: 19573611 PMC2733527

[23] Jenkinson M Smith S. A global optimisation method for robust affine registration of brain images. Med Image Anal. (2001) 5:143–56. doi: 10.1016/S1361-8415(01)00036-6, PMID: 11516708

[24] Adebimpe A Bertolero M Dolui S Cieslak M Murtha K Baller EB. ASLPrep: a platform for processing of arterial spin labeled MRI and quantification of regional brain perfusion. Nat Methods. (2022) 19:683–6. doi: 10.1038/s41592-022-01458-7, PMID: 35689029 PMC10548890

[25] Dolui S. Wolf R. L. Nabavizadeh S. A. Wolk D. A. Detre J. A. (2017). Automated quality evaluation index for 2D ASL CBF maps. International Society for Magnetic Resonance in Medicine; Honolulu, HI, USA. Available online at: https://archive.ismrm.org/2017/0682.html (Accessed July 30, 2024).

[26] Tustison NJ Cook PA Klein A Song G Das SR Duda JT. Large-scale evaluation of ANTs and free surfer cortical thickness measurements. Neuro Image. (2014) 99:166–79. doi: 10.1016/j.neuroimage.2014.05.044, PMID: 24879923

[27] Fischl B. Free surfer. Neuro Image. (2012) 62:774–81. doi: 10.1016/j.neuroimage.2012.01.021, PMID: 22248573 PMC3685476

[28] Cammoun L Gigandet X Meskaldji D Thiran JP Sporns O Do KQ. Mapping the human connectome at multiple scales with diffusion spectrum MRI. J Neurosci Methods. (2012) 203:386–97. doi: 10.1016/j.jneumeth.2011.09.031, PMID: 22001222

[29] Knopman DS Kramer JH Boeve BF Caselli RJ Graff-Radford NR Mendez MF. Development of methodology for conducting clinical trials in frontotemporal lobar degeneration. Brain. (2008) 131:2957–68. doi: 10.1093/brain/awn234, PMID: 18829698 PMC2725027

[30] Abe Y Kimura N Goto M Aso Y Matsubara E. Brain perfusion in Corticobasal syndrome with progressive aphasia. Dementia Geriatric Cogn Disord Extra. (2016) 6:133–41. doi: 10.1159/000443329 PMC4868931 27195001

[31] Katzdobler S Nitschmann A Barthel H Bischof G Beyer L Marek K. Additive value of [18F]PI-2620 perfusion imaging in progressive supranuclear palsy and corticobasal syndrome. Eur J Nucl Med Mol Imaging. (2023) 50:423–34. doi: 10.1007/s00259-022-05964-w, PMID: 36102964 PMC9816230

[32] Borroni B Perani D Agosti C Anchisi D Paghera B Archetti S. Tau haplotype influences cerebral perfusion pattern in frontotemporal lobar degeneration and related disorders. Acta Neurol Scand. (2008) 117:359–66. doi: 10.1111/j.1600-0404.2007.00955.x, PMID: 18177439

[33] Vandenberghe R. Classification of the primary progressive aphasias: principles and review of progress since 2011. Alzheimers Res Ther. (2016) 8:16. doi: 10.1186/s13195-016-0185-y, PMID: 27097664 PMC4839119

[34] Olm CA Peterson CS Irwin DJ Lee EB Trojanowski JQ Massimo L. Pathologic burden goes with the flow: MRI perfusion and pathologic burden in frontotemporal lobar degeneration due to tau. Imaging Neurosci. (2024) 2:1–12. doi: 10.1162/imag_a_00118 PMC12247599 40800459

[35] Bera G Migliaccio R Michelin T Lamari F Habert MO Dubois B. Distinct brain perfusion pattern associated with CSF biomarkers in semantic dementia. J Nucl Med. (2018) 66:271–3. doi: 10.3233/JAD-180087

[36] Ferraro PM Jester C Olm CA Placek K Agosta F Elman L. Perfusion alterations converge with patterns of pathological spread in transactive response DNA-binding protein 43 proteinopathies. Neurobiol Aging. (2018) 68:85–92. doi: 10.1016/j.neurobiolaging.2018.04.008, PMID: 29751289 PMC5993674

[37] Josephs KA Whitwell JL Knopman DS Boeve BF Vemuri P Senjem ML. Two distinct subtypes of right temporal variant frontotemporal dementia. Neurology. (2009) 73:1443–50. doi: 10.1212/WNL.0b013e3181bf9945, PMID: 19884571 PMC2779005

[38] Hu WT Wang Z Lee VM-Y Trojanowski JQ Detre JA Grossman M. Distinct cerebral perfusion patterns in FTLD and AD. Neurology. (2010) 75:881–8. doi: 10.1212/WNL.0b013e3181f11e35, PMID: 20819999 PMC2938974

[39] Seelaar H Papma JM Garraux G de Koning I Reijs AE Valkema R. Brain perfusion patterns in familial frontotemporal lobar degeneration. Neurology. (2011) 77:384–92. doi: 10.1212/WNL.0b013e3182270456, PMID: 21753175

[40] Tisdall MD Ohm DT Lobrovich R Das SR Mizsei G Prabhakaran K. Ex vivo MRI and histopathology detect novel iron-rich cortical inflammation in frontotemporal lobar degeneration with tau versus TDP-43 pathology. Neuro Image. (2022) 33:102913. doi: 10.1016/j.nicl.2021.102913, PMID: 34952351 PMC8715243

[41] Sarkar A Santiago RJ Smith R Kassaee A. Comparison of manual vs. automated multimodality (CT-MRI) image registration for brain tumors. Med Dosim. (2005) 30:20–4. doi: 10.1016/j.meddos.2004.10.004, PMID: 15749007

[42] Woods RP Grafton ST Watson JDG Sicotte NL Mazziotta JC. Automated image registration: II. Intersubject validation of linear and nonlinear models. J Comput Assist Tomogr. (1998) 22:153–65. doi: 10.1097/00004728-199801000-00028, PMID: 9448780

[43] Hu WT Rippon GW Boeve BF Knopman DS Petersen RC Parisi JE. Alzheimer’s disease and corticobasal degeneration presenting as corticobasal syndrome. Mov Disord. (2009) 24:1375–9. doi: 10.1002/mds.22574 19425061

[44] Dutt S Binney RJ Heuer HW Luong P Attygalle S Bhatt P. Progression of brain atrophy in PSP and CBS over 6 months and 1 year. Neurology. (2016) 87:2016–25. doi: 10.1212/WNL.0000000000003305, PMID: 27742814 PMC5109951

[45] Chen JJ Rosas HD Salat DH. Age-associated reductions in cerebral blood flow are independent from regional atrophy. Neuro Image. (2011) 55:468–78. doi: 10.1016/j.neuroimage.2010.12.032, PMID: 21167947 PMC3435846

[46] Sui J Adali T Yu Q Chen J Calhoun VD. A review of multivariate methods for multimodal fusion of brain imaging data. J Neurosci Methods. (2012) 204:68–81. doi: 10.1016/j.jneumeth.2011.10.031, PMID: 22108139 PMC3690333

