# Supplementary material for: Boundary-based registration improves sensitivity for detecting hypoperfusion in sporadic frontotemporal lobar degeneration
Source: Front Neurol. 2024 Aug 21;15:1452944. doi: 10.3389/fneur.2024.1452944 (PMC11371585; doi:10.3389/fneur.2024.1452944)
Supplement: Supplementary file 1 [file Table_1.docx]

**Supplementary Table 1:** **Significant Regions of Hypoperfusion.** Significant regions of hypoperfusion detected by FLIRT+BBR, manual+BBR, and manual registrations when comparing sFTLD-tau relative to controls, sFTLD-TDP relative to controls, and sFTLD-TDP relative to sFTLD-tau. Results are ordered in decreasing significance by t-statistic with region labels sourced from the Lausanne-125 parcellation.

|  | **FLIRT + BBR** | | | **Manual + BBR** | | | **Manual** | | |
| --- | --- | --- | --- | --- | --- | --- | --- | --- | --- |
| **sFTLD-Tau vs Controls** | Label Name | t | p | Label Name | t | p | Label Name | t | p |
|  | **left-caudalmiddlefrontal_3** | -6.712 | 6.439E-06 | **left-caudalmiddlefrontal_3** | -6.668 | 7.739E-06 | **left-caudalmiddlefrontal_3** | -7.239 | 4.458E-07 |
|  | **left-superiorfrontal_6** | -6.134 | 4.994E-05 | **left-caudalmiddlefrontal_1** | -6.072 | 8.582E-05 | **left-superiorfrontal_6** | -5.984 | 8.657E-05 |
|  | **left-caudalmiddlefrontal_1** | -6.106 | 7.547E-05 | **left-superiorfrontal_6** | -6.059 | 6.175E-05 | **left-caudalmiddlefrontal_1** | -5.895 | 1.424E-04 |
|  | **left-precentral_5** | -6.008 | 7.978E-05 | **left-precentral_5** | -5.983 | 8.542E-05 | **left-precentral_5** | -5.717 | 2.374E-04 |
|  | **left-superiorfrontal_5** | -5.518 | 4.505E-04 | **left-superiorfrontal_7** | -5.491 | 4.266E-04 | **left-superiorfrontal_9** | -5.253 | 1.109E-03 |
|  | **left-superiorfrontal_7** | -5.507 | 3.937E-04 | **left-superiorfrontal_5** | -5.490 | 4.960E-04 | **left-superiorfrontal_5** | -5.243 | 1.131E-03 |
|  | **left-superiorfrontal_4** | -5.192 | 1.560E-03 | **left-superiorfrontal_4** | -5.213 | 1.466E-03 | **left-superiorfrontal_7** | -5.158 | 1.206E-03 |
|  | **left-superiorfrontal_9** | -5.091 | 1.589E-03 | **left-superiorfrontal_9** | -5.104 | 1.498E-03 | **left-superiorfrontal_4** | -5.151 | 1.379E-03 |
|  | **right-caudalmiddlefrontal_3** | -4.773 | 4.451E-03 | **left-precentral_7** | -4.757 | 5.666E-03 | **right-superiorfrontal_8** | -4.831 | 2.473E-03 |
|  | **left-precentral_7** | -4.768 | 5.579E-03 | **right-caudalmiddlefrontal_3** | -4.728 | 5.164E-03 | **left-precentral_7** | -4.721 | 6.134E-03 |
|  | **left-parsopercularis_1** | -4.700 | 7.320E-03 | **left-superiorfrontal_3** | -4.681 | 7.782E-03 | **left-rostralmiddlefrontal_2** | -4.717 | 5.863E-03 |
|  | **right-superiorfrontal_8** | -4.684 | 4.934E-03 | **right-superiorfrontal_8** | -4.649 | 5.486E-03 | **left-parsopercularis_2** | -4.564 | 1.057E-02 |
|  | **left-rostralmiddlefrontal_1** | -4.677 | 8.356E-03 | **left-parsopercularis_1** | -4.642 | 8.728E-03 | **right-caudalmiddlefrontal_3** | -4.550 | 9.969E-03 |
|  | **left-superiorfrontal_3** | -4.673 | 7.962E-03 | **left-rostralmiddlefrontal_1** | -4.633 | 9.194E-03 | **left-superiorfrontal_3** | -4.507 | 1.485E-02 |
|  | **left-parsopercularis_2** | -4.492 | 1.564E-02 | **left-parsopercularis_2** | -4.488 | 1.545E-02 | **left-parsopercularis_1** | -4.350 | 2.242E-02 |
|  | **left-rostralmiddlefrontal_2** | -4.484 | 1.272E-02 | **left-rostralmiddlefrontal_2** | -4.456 | 1.402E-02 | **right-superiorfrontal_4** | -4.289 | 2.264E-02 |
|  | **left-superiorfrontal_8** | -4.252 | 2.371E-02 | **left-precentral_4** | -4.249 | 2.686E-02 | **left-rostralmiddlefrontal_1** | -4.272 | 2.767E-02 |
|  | **left-precentral_4** | -4.250 | 2.731E-02 | **left-superiorfrontal_8** | -4.248 | 2.415E-02 | **left-precentral_4** | -4.250 | 2.815E-02 |
|  | **left-precentral_3** | -4.215 | 2.906E-02 | **left-precentral_3** | -4.203 | 2.990E-02 | **left-superiorfrontal_2** | -4.220 | 3.687E-02 |
|  | **right-rostralmiddlefrontal_1** | -4.087 | 4.759E-02 | **right-superiorfrontal_5** | -4.089 | 3.653E-02 | **left-precentral_3** | -4.182 | 3.149E-02 |
|  | **right-superiorfrontal_5** | -4.080 | 3.772E-02 |  | | | **right-rostralmiddlefrontal_1** | -4.128 | 3.958E-02 |
|  | **left-rostralmiddlefrontal_4** | -4.059 | 4.470E-02 |  |  |  | **left-superiorfrontal_8** | -4.014 | 4.773E-02 |
| **sFTLD-TDP vs Controls** | **left-insula_3** | -9.289 | 1.070E-09 | **left-insula_3** | -9.136 | 1.824E-09 | **left-insula_3** | -9.461 | 1.640E-10 |
|  | **left-insula_2** | -8.831 | 8.150E-09 | **left-insula_2** | -8.638 | 1.799E-08 | **left-temporalpole_1** | -8.163 | 7.216E-07 |
|  | **left-fusiform_4** | -8.670 | 3.083E-08 | **left-temporalpole_1** | -8.617 | 8.928E-08 | **left-middletemporal_4** | -8.043 | 7.442E-07 |
|  | **left-temporalpole_1** | -8.659 | 9.323E-08 | **left-fusiform_4** | -8.476 | 5.120E-08 | **left-lateralorbitofrontal_2** | -7.671 | 8.001E-07 |
|  | **left-inferiortemporal_1** | -8.283 | 2.591E-07 | **left-middletemporal_4** | -8.207 | 4.785E-07 | **left-insula_2** | -7.626 | 2.320E-06 |
|  | **left-middletemporal_4** | -8.213 | 4.211E-07 | **left-inferiortemporal_1** | -8.192 | 2.995E-07 | **left-rostralanteriorcingulate_1** | -7.457 | 1.572E-06 |
|  | **left-rostralanteriorcingulate_1** | -7.855 | 2.262E-07 | **left-rostralanteriorcingulate_1** | -8.029 | 1.067E-07 | **left-medialorbitofrontal_2** | -7.311 | 7.011E-06 |
|  | **right-insula_2** | -7.822 | 6.810E-08 | **left-medialorbitofrontal_2** | -7.969 | 8.836E-07 | **left-superiortemporal_5** | -7.153 | 9.305E-06 |
|  | **left-middletemporal_3** | -7.692 | 6.483E-06 | **right-insula_2** | -7.786 | 5.535E-08 | **left-middletemporal_3** | -6.951 | 7.454E-05 |
|  | **left-medialorbitofrontal_2** | -7.610 | 3.294E-06 | **left-middletemporal_3** | -7.491 | 1.221E-05 | **right-insula_2** | -6.899 | 3.842E-06 |
|  | **left-lateralorbitofrontal_2** | -7.523 | 1.561E-06 | **left-lateralorbitofrontal_2** | -7.427 | 2.060E-06 | **left-inferiortemporal_1** | -6.884 | 4.746E-05 |
|  | **left-superiortemporal_5** | -7.517 | 3.231E-06 | **left-superiortemporal_5** | -7.332 | 6.321E-06 | **left-superiortemporal_4** | -6.748 | 1.161E-05 |
|  | **right-insula_3** | -7.202 | 7.615E-06 | **right-insula_3** | -7.152 | 7.479E-06 | **left-inferiortemporal_3** | -6.637 | 2.541E-05 |
|  | **left-insula_4** | -6.754 | 1.627E-05 | **left-inferiortemporal_3** | -6.608 | 3.227E-05 | **right-posteriorcingulate_1** | -6.374 | 1.266E-05 |
|  | **left-inferiortemporal_3** | -6.676 | 2.499E-05 | **left-medialorbitofrontal_1** | -6.601 | 8.921E-05 | **left-medialorbitofrontal_1** | -6.260 | 2.787E-04 |
|  | **left-middletemporal_2** | -6.528 | 3.486E-04 | **right-middletemporal_3** | -6.553 | 9.082E-05 | **left-fusiform_4** | -6.251 | 1.367E-04 |
|  | **right-middletemporal_3** | -6.507 | 1.243E-04 | **left-superiorfrontal_2** | -6.506 | 2.238E-04 | **left-superiorfrontal_3** | -6.244 | 2.922E-04 |
|  | **left-superiortemporal_4** | -6.492 | 4.850E-05 | **left-insula_4** | -6.394 | 5.309E-05 | **left-lateralorbitofrontal_1** | -6.224 | 2.014E-04 |
|  | **left-parsopercularis_2** | -6.486 | 8.743E-05 | **right-posteriorcingulate_1** | -6.380 | 2.004E-05 | **right-temporalpole_1** | -6.222 | 2.344E-04 |
|  | **left-medialorbitofrontal_1** | -6.399 | 1.765E-04 | **right-temporalpole_1** | -6.359 | 1.673E-04 | **right-insula_3** | -6.146 | 3.175E-04 |
|  | **left-lateralorbitofrontal_1** | -6.389 | 1.307E-04 | **left-lateralorbitofrontal_1** | -6.327 | 1.531E-04 | **left-insula_1** | -6.116 | 4.935E-05 |
|  | **left-superiorfrontal_2** | -6.368 | 3.260E-04 | **left-superiortemporal_4** | -6.281 | 1.099E-04 | **right-middletemporal_3** | -6.107 | 4.390E-04 |
|  | **right-temporalpole_1** | -6.341 | 1.747E-04 | **left-superiorfrontal_3** | -6.261 | 2.517E-04 | **left-bankssts_1** | -6.067 | 8.222E-05 |
|  | **left-superiorfrontal_3** | -6.258 | 2.513E-04 | **left-middletemporal_2** | -6.252 | 6.772E-04 | **left-middletemporal_2** | -6.028 | 1.037E-03 |
|  | **right-posteriorcingulate_1** | -6.228 | 3.488E-05 | **left-parsopercularis_2** | -6.154 | 2.613E-04 | **left-parsopercularis_2** | -5.956 | 3.361E-04 |
|  | **right-middletemporal_4** | -6.168 | 2.183E-04 | **left-supramarginal_3** | -6.085 | 6.247E-05 | **left-superiorfrontal_2** | -5.840 | 1.289E-03 |
|  | **left-parsorbitalis_1** | -6.137 | 2.819E-04 | **right-middletemporal_4** | -6.084 | 2.785E-04 | **left-insula_4** | -5.840 | 2.005E-04 |
|  | **left-bankssts_2** | -6.040 | 3.716E-04 | **left-bankssts_2** | -6.020 | 3.319E-04 | **left-parstriangularis_1** | -5.823 | 4.192E-04 |
|  | **left-parstriangularis_1** | -6.031 | 2.969E-04 | **left-caudalanteriorcingulate_1** | -5.950 | 3.513E-04 | **left-supramarginal_3** | -5.822 | 2.090E-04 |
|  | **left-inferiortemporal_2** | -6.029= | 2.578E-041 | **right-lateralorbitofrontal_2** | -5.935 | 1.009E-04 | **right-middletemporal_4** | -5.783 | 9.083E-04 |
|  | **left-supramarginal_3** | -6.029 | 9.256E-05 | **left-parsorbitalis_1** | -5.918 | 6.171E-04 | **left-inferiortemporal_2** | -5.727 | 3.942E-04 |
|  | **left-posteriorcingulate_2** | -5.885 | 2.423E-04 | **left-parstriangularis_1** | -5.906 | 4.346E-04 | **left-bankssts_2** | -5.632 | 5.664E-04 |
|  | **right-lateralorbitofrontal_2** | -5.864 | 1.431E-04 | **left-inferiortemporal_2** | -5.845 | 4.572E-04 | **left-superiorfrontal_1** | -5.557 | 2.276E-03 |
|  | **left-parsopercularis_1** | -5.860 | 7.699E-04 | **left-parsopercularis_1** | -5.790 | 9.230E-04 | **left-parsopercularis_1** | -5.508 | 1.881E-03 |
|  | **left-insula_1** | -5.835 | 3.308E-04 | **left-posteriorcingulate_2** | -5.782 | 3.793E-04 | **left-precentral_8** | -5.401 | 1.814E-03 |
|  | **left-caudalanteriorcingulate_1** | -5.833 | 5.578E-041 | **right-superiortemporal_5** | -5.716 | 8.895E-04 | **left-parsorbitalis_1** | -5.363 | 2.705E-03 |
|  | **right-superiortemporal_5** | -5.721 | 8.428E-04 | **left-bankssts_1** | -5.673 | 7.963E-04 | **left-superiorfrontal_5** | -5.352 | 1.896E-03 |
|  | **left-bankssts_1** | -5.717 | 7.967E-04 | **left-superiorfrontal_1** | -5.619 | 1.781E-03 | **left-lingual_3** | -5.329 | 6.971E-04 |
|  | **left-supramarginal_5** | -5.709 | 4.366E-04 | **left-precentral_8** | -5.612 | 1.046E-03 | **left-posteriorcingulate_2** | -5.285 | 2.444E-03 |
|  | **left-precentral_8** | -5.688 | 8.015E-04 | **left-supramarginal_5** | -5.553 | 0.000745671153548965 | **right-superiortemporal_5** | -5.230 | 4.578E-03 |
|  | **left-superiorfrontal_1** | -5.564 | 2.146E-03 | **left-insula_1** | -5.479 | 0.00096373790780437 | **left-inferiorparietal_3** | -5.202 | 4.357E-03 |
|  | **left-superiorfrontal_7** | -5.382 | 1.592E-03 | **left-superiorfrontal_7** | -5.381 | 1.627E-03 | **right-lateralorbitofrontal_2** | -5.201 | 1.856E-03 |
|  | **left-superiorfrontal_5** | -5.380 | 2.123E-03 | **right-medialorbitofrontal_2** | -5.318 | 4.949E-03 | **left-supramarginal_5** | -5.085 | 3.934E-03 |
|  | **right-rostralanteriorcingulate_1** | -5.285 | 3.924E-03 | **left-superiorfrontal_5** | -5.288 | 2.815E-03 | **right-superiorfrontal_3** | -5.068 | 5.584E-03 |
|  | **right-superiorfrontal_3** | -5.251 | 2.707E-03 | **right-paracentral_3** | -5.268 | 0.000699268457686873 | **right-rostralanteriorcingulate_1** | -5.066 | 3.869E-03 |
|  | **right-medialorbitofrontal_2** | -5.244 | 5.677E-03 | **left-superiorfrontal_4** | -5.254 | 3.734E-03 | **right-medialorbitofrontal_2** | -5.033 | 6.595E-03 |
|  | **right-insula_1** | -5.229 | 3.372E-03 | **left-lateralorbitofrontal_3** | -5.249 | 3.326E-03 | **right-lingual_2** | -5.026 | 2.928E-03 |
|  | **left-superiorfrontal_4** | -5.174 | 4.665E-03 | **right-rostralanteriorcingulate_1** | -5.236 | 4.587E-03 | **left-superiortemporal_3** | -5.020 | 8.060E-03 |
|  | **left-superiortemporal_3** | -5.164 | 4.713E-03 | **right-superiorfrontal_3** | -5.192 | 3.315E-03 | **left-superiorfrontal_9** | -5.016 | 4.873E-03 |
|  | **right-parstriangularis_1** | -5.155 | 2.519E-03 | **right-parstriangularis_1** | -5.187 | 2.388E-03 | **right-fusiform_4** | -5.011 | 6.977E-03 |
|  | **left-lateralorbitofrontal_3** | -5.152 | 4.579E-03 | **right-lateralorbitofrontal_3** | -5.181 | 1.057E-03 | **left-middletemporal_1** | -5.002 | 6.307E-03 |
|  | **left-inferiorparietal_3** | -5.152 | 6.296E-03 | **right-insula_1** | -5.176 | 3.495E-03 | **left-paracentral_2** | -4.987 | 2.895E-03 |
|  | **right-middletemporal_2** | -5.150 | 3.984E-03 | **right-middletemporal_2** | -5.143 | 3.863E-03 | **left-inferiorparietal_5** | -4.968 | 1.527E-02 |
|  | **right-parahippocampal_1** | -5.136 | 3.898E-03 | **right-lingual_3** | -5.134 | 1.876E-03 | **right-parahippocampal_1** | -4.946 | 6.781E-03 |
|  | **right-lingual_3** | -5.111 | 1.998E-033 | **right-parahippocampal_1** | -5.114 | 4.351E-03 | **left-precuneus_5** | -4.944 | 4.188E-03 |
|  | **right-parsorbitalis_1** | -5.080 | 3.048E-03 | **right-superiortemporal_4** | -5.111 | 2.940E-03 | **right-paracentral_3** | -4.942 | 2.761E-03 |
|  | **right-paracentral_3** | -5.079 | 1.427E-03 | **right-lingual_2** | -5.108 | 2.014E-03 | **left-caudalanteriorcingulate_1** | -4.919 | 1.045E-02 |
|  | **right-lateralorbitofrontal_3** | -5.058 | 1.677E-03 | **left-inferiorparietal_3** | -5.074 | 6.916E-03 | **right-superiorfrontal_7** | -4.895 | 4.295E-03 |
|  | **right-superiortemporal_3** | -5.058 | 3.167E-03 | **right-fusiform_4** | -5.039 | 4.933E-03 | **right-parstriangularis_1** | -4.892 | 4.849E-03 |
|  | **right-lingual_2** | -5.038 | 2.560E-03 | **left-frontalpole_1** | -5.035 | 6.241E-03 | **left-inferiortemporal_4** | -4.877 | 1.162E-02 |
|  | **right-fusiform_4** | -5.030 | 4.757E-03 | **right-superiortemporal_3** | -5.022 | 3.504E-03 | **left-supramarginal_2** | -4.760 | 1.243E-02 |
|  | **right-medialorbitofrontal_1** | -5.030 | 7.258E-03 | **left-superiortemporal_3** | -5.018 | 7.176E-03 | **left-superiorfrontal_4** | -4.728 | 1.666E-02 |
|  | **left-middletemporal_1** | -5.013 | 7.184E-03 | **right-medialorbitofrontal_1** | -5.011 | 7.886E-03 | **right-lingual_3** | -4.727 | 7.369E-03 |
|  | **right-superiorfrontal_7** | -5.013 | 2.243E-03 | **right-superiorfrontal_7** | -4.970 | 2.761E-03 | **left-superiorfrontal_7** | -4.725 | 1.322E-02 |
|  | **right-supramarginal_3** | -4.984 | 2.916E-03 | **right-pericalcarine_1** | -4.968 | 4.243E-03 | **left-caudalmiddlefrontal_1** | -4.695 | 1.833E-02 |
|  | **left-parahippocampal_1** | -4.963 | 6.796E-03 | **right-superiorfrontal_6** | -4.963 | 1.913E-03 | **right-pericalcarine_1** | -4.683 | 1.021E-02 |
|  | **right-pericalcarine_1** | -4.959 | 4.209E-03 | **left-lingual_3** | -4.941 | 2.616E-03 | **left-supramarginal_4** | -4.679 | 1.946E-02 |
|  | **left-frontalpole_1** | -4.956 | 8.207E-03 | **right-parsorbitalis_1** | -4.936 | 5.006E-03 | **left-frontalpole_1** | -4.679 | 1.659E-02 |
|  | **left-lingual_3** | -4.948 | 2.422E-03 | **left-parahippocampal_1** | -4.934 | 7.344E-03 | **left-supramarginal_1** | -4.667 | 8.602E-03 |
|  | **left-superiorfrontal_6** | -4.941 | 1.230E-02 | **left-superiorfrontal_6** | -4.912 | 1.286E-02 | **right-superiorfrontal_6** | -4.661 | 6.836E-03 |
|  | **right-superiortemporal_4** | -4.935 | 5.108E-03 | **left-middletemporal_1** | -4.893 | 9.286E-03 | **right-paracentral_2** | -4.643 | 5.782E-03 |
|  | **left-supramarginal_2** | -4.878 | 9.335E-03 | **left-supramarginal_2** | -4.862 | 9.221E-03 | **right-precentral_6** | -4.642 | 4.227E-03 |
|  | **right-superiorfrontal_6** | -4.875 | 2.632E-03 | **right-superiorfrontal_2** | -4.836 | 9.653E-03 | **right-middletemporal_2** | -4.611 | 2.134E-02 |
|  | **left-paracentral_2** | -4.862 | 5.238E-03 | **right-precentral_6** | -4.832 | 2.249E-03 | **left-superiorparietal_2** | -4.610 | 9.979E-03 |
|  | **right-precuneus_1** | -4.861 | 4.716E-03 | **right-precuneus_1** | -4.831 | 5.840E-03 | **right-parsorbitalis_1** | -4.609 | 1.305E-02 |
|  | **right-superiorfrontal_2** | -4.832 | 9.587E-03 | **left-paracentral_2** | -4.828 | 5.975E-03 | **right-superiortemporal_3** | -4.602 | 1.468E-02 |
|  | **right-precentral_6** | -4.820 | 2.360E-03 | **right-paracentral_2** | -4.825 | 3.334E-03 | **right-middletemporal_1** | -4.574 | 1.156E-02 |
|  | **left-inferiorparietal_5** | -4.813 | 2.410E-02 | **right-superiorfrontal_5** | -4.800 | 5.946E-03 | **right-superiorfrontal_2** | -4.573 | 2.104E-02 |
|  | **right-superiorfrontal_5** | -4.768 | 6.642E-03 | **right-supramarginal_3** | -4.787 | 5.339E-03 | **right-superiorfrontal_5** | -4.571 | 1.344E-02 |
|  | **left-inferiortemporal_4** | -4.768 | 1.563E-02 | **left-inferiortemporal_4** | -4.784 | 1.540E-02 | **right-posteriorcingulate_2** | -4.548 | 1.597E-02 |
|  | **right-fusiform_3** | -4.717 | 9.332E-03 | **left-inferiorparietal_5** | -4.776 | 2.615E-02 | **right-cuneus_1** | -4.516 | 1.124E-02 |
|  | **right-superiorfrontal_4** | -4.714 | 1.127E-02 | **right-inferiortemporal_2** | -4.748 | 1.438E-02 | **left-isthmuscingulate_1** | -4.510 | 3.720E-02 |
|  | **left-rostralmiddlefrontal_6** | -4.712 | 2.254E-02 | **right-superiortemporal_2** | -4.743 | 5.260E-03 | **left-superiorfrontal_6** | -4.499 | 3.800E-02 |
|  | **right-paracentral_2** | -4.677 | 5.750E-03 | **left-precentral_7** | -4.711 | 1.358E-02 | **left-lateralorbitofrontal_3** | -4.497 | 2.909E-02 |
|  | **right-middletemporal_1** | -4.674 | 7.652E-03 | **right-fusiform_3** | -4.701 | 1.000E-02 | **right-inferiortemporal_3** | -4.493 | 2.164E-02 |
|  | **right-inferiortemporal_2** | -4.663 | 1.708E-02 | **left-rostralmiddlefrontal_6** | -4.667 | 2.544E-02 | **right-insula_1** | -4.459 | 3.276E-02 |
|  | **right-superiortemporal_2** | -4.645 | 7.636E-03 | **right-superiorfrontal_4** | -4.654 | 1.361E-02 | **left-superiorfrontal_8** | -4.428 | 1.427E-02 |
|  | **right-parsopercularis_2** | -4.636 | 1.874E-02 | **left-supramarginal_1** | -4.642 | 1.011E-02 | **right-precentral_1** | -4.426 | 3.082E-02 |
|  | **left-caudalmiddlefrontal_1** | -4.628 | 2.763E-02 | **left-superiorfrontal_9** | -4.641 | 1.391E-02 | **right-medialorbitofrontal_1** | -4.422 | 3.210E-02 |
|  | **left-supramarginal_1** | -4.619 | 1.165E-02 | **left-caudalmiddlefrontal_1** | -4.623 | 2.787E-02 | **left-precentral_7** | -4.409 | 3.111E-02 |
|  | **right-superiorfrontal_8** | -4.615 | 1.141E-02 | **right-precuneus_3** | -4.594 | 2.151E-02 | **right-superiorfrontal_1** | -4.403 | 4.655E-02 |
|  | **right-precentral_1** | -4.600 | 1.739E-02 | **right-parsopercularis_2** | -4.592 | 2.090E-02 | **left-parahippocampal_1** | -4.389 | 4.260E-02 |
|  | **left-precentral_7** | -4.584 | 2.090E-02 | **right-precentral_1** | -4.582 | 1.795E-02 | **right-lateralorbitofrontal_3** | -4.378 | 1.400E-02 |
|  | **right-precuneus_3** | -4.577 | 2.181E-02 | **left-superiorparietal_1** | -4.571 | 6.688E-03 | **right-superiortemporal_4** | -4.371 | 2.632E-02 |
|  | **right-entorhinal_1** | -4.575 | 2.126E-02 | **right-posteriorcingulate_2** | -4.569 | 1.736E-02 | **left-rostralmiddlefrontal_2** | -4.365 | 3.597E-02 |
|  | **left-superiorparietal_1** | -4.573 | 6.575E-03 | **right-middletemporal_1** | -4.560 | 1.022E-02 | **right-inferiortemporal_1** | -4.355 | 4.337E-02 |
|  | **left-precuneus_5** | -4.557 | 1.925E-02 | **right-superiorfrontal_8** | -4.527 | 1.480E-02 | **left-postcentral_3** | -4.331 | 2.962E-02 |
|  | **left-superiorfrontal_9** | -4.525 | 2.075E-02 | **right-isthmuscingulate_1** | -4.524 | 2.298E-02 | **right-supramarginal_3** | -4.323 | 2.546E-02 |
|  | **right-isthmuscingulate_1** | -4.482 | 2.560E-02 | **right-precuneus_2** | -4.502 | 1.722E-02 | **right-fusiform_3** | -4.319 | 3.038E-02 |
|  | **right-lateralorbitofrontal_1** | -4.476 | 2.380E-02 | **right-lateralorbitofrontal_1** | -4.493 | 2.294E-02 | **right-inferiortemporal_2** | -4.316 | 3.271E-02 |
|  | **right-precuneus_2** | -4.466 | 1.886E-02 | **right-parstriangularis_2** | -4.476 | 2.657E-02 | **right-superiorfrontal_8** | -4.217 | 3.964E-02 |
|  | **left-isthmuscingulate_1** | -4.435 | 4.859E-02 | **left-precuneus_5** | -4.475 | 2.493E-02 | **right-superiortemporal_2** | -4.196 | 3.304E-02 |
|  | **right-superiorfrontal_1** | -4.426 | 4.896E-02 | **right-entorhinal_1** | -4.462 | 3.014E-02 | **left-superiorparietal_1** | -4.093 | 3.119E-02 |
|  | **left-rostralmiddlefrontal_5** | -4.418 | 4.602E-02 | **right-superiorfrontal_1** | -4.434 | 4.814E-02 |  | | |
|  | **left-superiorfrontal_8** | -4.417 | 1.734E-02 | **left-postcentral_4** | -4.432 | 2.549E-02 |  |  |  |
|  | **right-parstriangularis_2** | -4.398 | 3.550E-02 | **right-medialorbitofrontal_3** | -4.382 | 4.798E-02 |  |  |  |
|  | **right-posteriorcingulate_2** | -4.387 | 3.027E-02 | **right-inferiortemporal_1** | -4.378 | 4.037E-02 |  |  |  |
|  | **left-rostralmiddlefrontal_2** | -4.380 | 3.589E-02 | **left-rostralmiddlefrontal_2** | -4.370 | 3.678E-02 |  |  |  |
|  | **left-postcentral_4** | -4.379 | 2.952E-02 | **right-cuneus_1** | -4.369 | 1.876E-02 |  |  |  |
|  | **right-rostralmiddlefrontal_1** | -4.350 | 3.289E-02 | **left-superiorfrontal_8** | -4.358 | 2.151E-02 |  |  |  |
|  | **right-rostralmiddlefrontal_4** | -4.343 | 3.300E-02 | **left-superiorparietal_2** | -4.348 | 2.571E-02 |  |  |  |
|  | **right-inferiortemporal_1** | -4.320 | 4.853E-02 | **right-rostralmiddlefrontal_1** | -4.319 | 3.569E-02 |  |  |  |
|  | **right-precentral_4** | -4.311 | 1.797E-02 | **right-inferiortemporal_4** | -4.291 | 2.645E-02 |  |  |  |
|  | **left-rostralmiddlefrontal_3** | -4.309 | 4.667E-02 | **right-transversetemporal_1** | -4.286 | 2.706E-02 |  |  |  |
|  | **left-superiorparietal_2** | -4.303 | 3.079E-02 | **right-precentral_4** | -4.285 | 1.934E-02 |  |  |  |
|  | **right-cuneus_1** | -4.300 | 2.325E-02 | **left-postcentral_3** | -4.242 | 4.222E-02 |  |  |  |
|  | **right-transversetemporal_1** | -4.295 | 2.680E-02 | **right-rostralmiddlefrontal_4** | -4.239 | 4.433E-02 |  |  |  |
|  | **right-inferiortemporal_3** | -4.281 | 4.055E-02 | **right-inferiortemporal_3** | -4.239 | 4.486E-02 |  |  |  |
|  | **left-postcentral_3** | -4.249 | 4.231E-02 | **right-caudalmiddlefrontal_3** | -4.233 | 4.859E-02 |  |  |  |
|  | **right-inferiortemporal_4** | -4.219 | 3.415E-02 |  | | |  |  |  |
|  | **left-precuneus_1** | -4.196 | 4.505E-02 |  |  |  |  |  |  |
| **sFTLD-TDP vs sFTLD-tau** | **left-insula_2** | 4.223 | 4.329E-02 | **left-insula_2** | 4.205 | 4.461E-02 | **left-insula_3** | 4.445 | 2.517E-02 |
|  | **left-middletemporal_3** | 4.319 | 2.993E-02 | **left-middletemporal_3** | 4.245 | 3.759E-02 | **left-middletemporal_4** | 4.700 | 1.004E-02 |
|  | **left-middletemporal_4** | 4.744 | 9.123E-03 | **left-insula_3** | 4.709 | 1.011E-02 | **left-temporalpole_1** | 4.746 | 8.648E-03 |
|  | **left-insula_3** | 4.772 | 8.449E-03 | **left-middletemporal_4** | 4.721 | 9.674E-03 |  | | |
|  | **left-temporalpole_1** | 4.930 | 5.038E-03 | **left-temporalpole_1** | 4.852 | 6.313E-03 |  |  |  |
