# Supplementary material for: Boundary-based registration improves sensitivity for detecting hypoperfusion in sporadic frontotemporal lobar degeneration
Source: Front Neurol. 2024 Aug 21;15:1452944. doi: 10.3389/fneur.2024.1452944 (PMC11371585; doi:10.3389/fneur.2024.1452944)
Supplement: Supplementary file 2 [file Table_2.DOCX]

Code that was used to perform CBF-T1w registrations is available in a public repository (<https://github.com/ftdc-picsl/hcpASLregInTauTDP>).

FTDHCP imaging data is available in a NIMH Data Archive (<https://nda.nih.gov/edit_collection.html?id=3160>).
